# Evidence for widespread human exposure to food contact chemicals

**DOI:** 10.1038/s41370-024-00718-2

**Published:** 2024-09-17

**Authors:** Birgit Geueke, Lindsey V. Parkinson, Ksenia J. Groh, Christopher D. Kassotis, Maricel V. Maffini, Olwenn V. Martin, Lisa Zimmermann, Martin Scheringer, Jane Muncke

**Affiliations:** 1 Food Packaging Forum Foundation, Zurich, Switzerland; 2 Department of Environmental Toxicology, Eawag, Swiss Federal Institute of Aquatic Science and Technology, Duebendorf, Switzerland; 3 Institute of Environmental Health Sciences and Department of Pharmacology, Wayne State University, Detroit, MI USA; 4 Independent Consultant, Frederick, MD USA; 5 Department of Arts & Science, Plastic Waste Innovation Hub, University College London, London, UK; 6 RECETOX, Masaryk University, Brno, Czech Republic; 7 Department of Environmental Systems Science, ETH Zurich, Zurich, Switzerland

## Abstract

**Background:**

Over 1800 food contact chemicals (FCCs) are known to migrate from food contact articles used to store, process, package, and serve foodstuffs. Many of these FCCs have hazard properties of concern, and still others have never been tested for toxicity. Humans are known to be exposed to FCCs via foods, but the full extent of human exposure to all FCCs is unknown.

**Objective:**

To close this important knowledge gap, we conducted a systematic overview of FCCs that have been monitored and detected in human biomonitoring studies according to a previously published protocol.

**Methods:**

We first compared the more than 14,000 known FCCs to five biomonitoring programs and three metabolome/exposome databases. In a second step, we prioritized FCCs that have been frequently detected in food contact materials and systematically mapped the available evidence for their presence in humans.

**Results:**

For 25% of the known FCCs (3601), we found evidence for their presence in humans. This includes 194 FCCs from human biomonitoring programs, with 80 of these having hazard properties of high concern. Of the 3528 FCCs included in metabolome/exposome databases, most are from the Blood Exposome Database. We found evidence for the presence in humans for 63 of the 175 prioritized FCCs included in the systematic evidence map, and 59 of the prioritized FCCs lack hazard data.

**Significance:**

Notwithstanding that there are also other sources of exposure for many FCCs, these data will help to prioritize FCCs of concern by linking information on migration and biomonitoring. Our results on FCCs monitored in humans are available as an interactive dashboard (FCChumon) to enable policymakers, public health researchers, and food industry decision-makers to make food contact materials and articles safer, reduce human exposure to hazardous FCCs and improve public health.

**Impact statement:**

We present systematically compiled evidence on human exposure to 3601 food contact chemicals (FCCs) and highlight FCCs that are of concern because of their known hazard properties. Further, we identify relevant data gaps for FCCs found in food contact materials and foods. This article improves the understanding of food contact materials’ contribution to chemical exposure for the human population and highlights opportunities for improving public health.

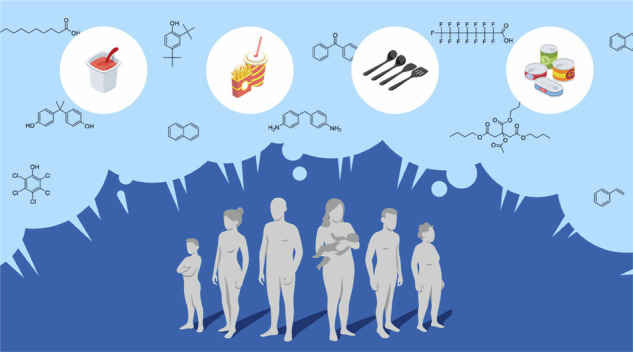

## Introduction

Humans are exposed to synthetic chemicals from food, drugs, household and personal care products, and environmental pollutants. Some of these chemicals have been associated with the increasing prevalence of non-communicable diseases [1–3]. Food packaging and other food contact articles (FCAs), such as tableware and food processing equipment, contribute to the human chemical burden via oral exposure, because food contact chemicals (FCCs) migrate from different food contact materials (FCMs) into foodstuffs and are then ingested [4–8].

For individual FCCs, such as bisphenol A (BPA) and several phthalates, the contribution of chemical migration from FCMs to human exposure has been studied in detail, taking into account that other exposure sources exist [9–12]. BPA is banned in some food contact applications, such as baby bottles, in many parts of the world, but is still regularly measured in FCMs (e.g [13–15].). Currently, a complete ban on BPA in FCMs is proposed by the European Commission [16]. However, hundreds of FCCs have been shown to migrate from FCMs into foods, and thousands of FCCs have been extracted from FCMs [5]. In total, over 12,000 FCCs could be intentionally used during the manufacturing of different types of FCMs [17] and even more chemicals could be present in FCMs as non-intentionally added substances (NIAS) that are introduced or formed during manufacture or use [5, 18, 19].

Many FCCs are of concern for human health because they have hazard properties such as carcinogenicity, mutagenicity, and reprotoxicity (CMR), endocrine disrupting properties, bioaccumulation potential, and/or persistence [17, 20, 21]. In addition, toxicity data are often incomplete or missing, which means that safe use cannot be assessed [17, 22, 23]. Therefore, reducing exposure to known hazardous FCCs and assessing untested FCCs can contribute to the prevention of non-communicable diseases that are associated with chemical exposures [24, 25].

The challenges in regulating FCMs and managing the health risks associated with FCCs are diverse and legislation often does not keep up with the latest scientific understanding [26, 27]. Publicly available evidence on intentionally used FCCs and their known hazards is available in our earlier work where we compiled the Food Contact Chemicals Database (FCCdb) [17]. The FCCdb gives an overview of all chemicals that are known to be used in the manufacture of FCMs. Further, we systematically mapped data on migrating and extractable FCCs, and our Database on Migrating and Extractable Food Contact Chemicals (FCCmigex) provides evidence for FCCs that have been detected in extracts of FCMs and/or their migrates into food and food simulants, indicating the potential for human exposure [5]. Only 30% of the chemicals present in FCMs are listed in the FCCdb, based on information from the most recent update of the FCCmigex database [28]. This indicates that the non-listed FCCs are either NIAS or have been intentionally used although they are not recorded in any of the FCCdb’s sources. Even though it is well-established that chemicals migrating from FCMs contribute to human exposure, the presence of FCCs in human samples has not yet been systematically assessed.

Here, we provide a systematic overview of FCCs that have been monitored and detected in humans by including information from biomonitoring programs, metabolome and exposome databases, and the primary scientific literature. We detailed our approach in a previously published protocol [29]. The resulting Database on Food Contact Chemicals Monitored in Humans (FCChumon) is a publicly available tool integrating empirical data on FCCs in human samples, and it complements the FCCdb and FCCmigex databases. Our goal is to provide scientific evidence that supports advancing global FCM regulations and the safety assessments of FCCs.

## Methods

### Overview of the two-step approach

The evidence for FCCs that have been monitored and detected in human samples was compiled according to a protocol initially registered on Zenodo in September 2022 and updated in April 2023 [29]. We followed the structure of a Population-Outcome (PO) question:

- Question: Which known FCCs have been monitored in the human body?
- Population (P): Human samples, such as blood, urine, hair, and breast milk, from people of any age, gender, or ethnicity
- Outcome (O): Any result describing the monitoring/detection of a known FCC or its metabolite

**Fig. 1:**
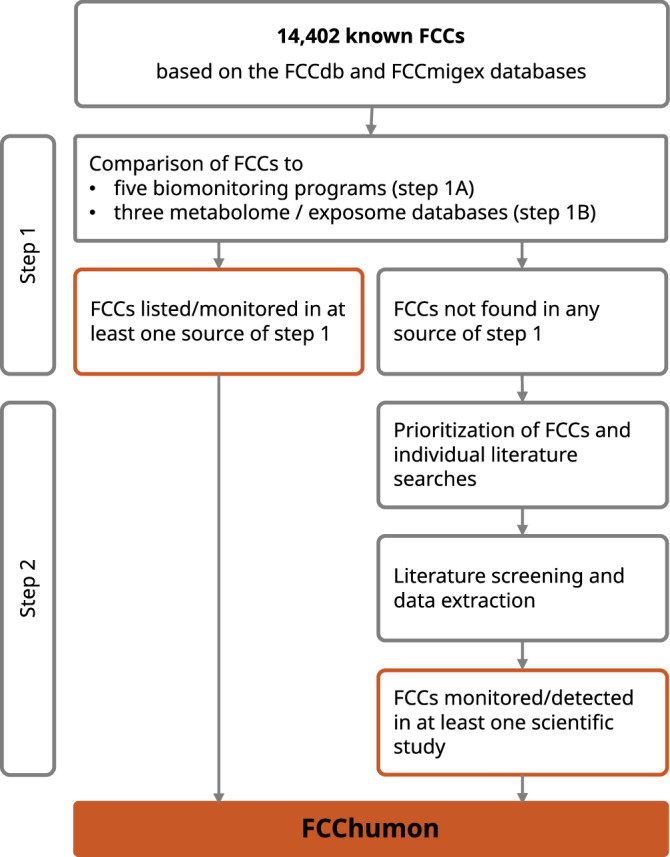
Overview of the stepwise strategy for identifying FCCs with evidence for human exposure. We compared known FCCs to biomonitoring programs and metabolome/exposome databases (step 1) and systematically mapped the evidence for presence of additional, priority FCCs in humans (step 2). The results of steps 1 and step 2 (red boxes) comprise the Database of Food Contact Chemicals Monitored in Humans (FCChumon).

As detailed in the protocol and further specified below, we applied a stepwise approach and referred to biomonitoring programs, databases on the human exposome and metabolome, and the primary scientific literature to map the evidence for FCCs’ presence in humans. Briefly, in step 1, FCCs included in the FCCdb and the FCCmigex databases were matched to the chemicals listed in biomonitoring programs and metabolome and exposome databases (Fig. 1). During protocol development, we found that thousands of FCCs were neither included in the selected metabolome/exposome databases nor in biomonitoring programs, while the primary scientific literature reported the monitoring of some of these FCCs in human samples. In step 2, we therefore applied the methodology of a systematic evidence map to obtain relevant information from the scientific literature. FCCs not found in any of the sources consulted in step 1 were prioritized based on their presence in FCMs, according to evidence from FCCmigex. These prioritized FCCs were included in the systematic evidence mapping performed in step 2 to understand their presence in human samples.

### Information sources for chemical comparisons

Together, the FCCdb and the FCCmigex databases presently consist of 14,402 known FCCs with assigned CAS Registry Numbers (Fig. 1). The FCCdb is an inventory for FCCs that are potentially used in the manufacture of FCMs and FCAs [17]. It currently contains 12,285 distinct FCCs of which 11,593 have a CAS Registry Number. The FCCmigex database systematically maps scientific evidence of FCCs that have been measured in FCMs and FCAs [5, 28]. The most recent version of the FCCmigex database contains 4262 chemicals with a CAS Registry Number, of which 3995 FCCs have been detected at least once in an FCM migrate or extract. Each FCCmigex database entry is linked to the reference from which it was generated and provides information about the FCC, what type of FCA and which FCM(s) were tested, details about the experimental set-up, and whether the FCC was detected or not. Chemicals that have been targeted but never detected in FCMs, and that are not in the FCCdb, are not included in this study.

In the first step, we consulted five biomonitoring programs that encompass different ranges of chemicals and provide wide geographic coverage, namely the National Health and Nutrition Examination Survey (NHANES) of the US [30], the Canadian Health Measures Survey (CHMS) [31], the Human Biomonitoring for Europe project (HBM4EU) [32, 33], the Korean National Environmental Health Survey (KoNEHS) [34], and Biomonitoring California [35]. Further, three metabolome/exposome databases were used to identify FCCs that have been monitored in humans: the Human Metabolome Database (HMDB) [36, 37]; the Blood Exposome Database [38, 39], and the Exposome Explorer [40, 41]. In addition to these sources, in the second step we systematically searched the primary scientific literature for human biomonitoring data on specific FCCs, using bibliographic databases (PubMed, Web of Science Core Collection (WoS), ScienceDirect, and CAS SciFinder^n^).

### Data processing and comparisons (step 1)

All known FCCs with CAS Registry Numbers were included in the comparisons of step 1, regardless of whether the CAS Registry Number indicates a specific structure or a chemical mixture. If available, additional chemical identifiers, such as INChI Keys and SMILES, were retrieved from the collections of FCCs associated with lists S77 and S112 from the NORMAN Suspect List Exchange [42–44].

In step 1A, information on chemicals that are part of any of the biomonitoring programs was downloaded from the respective sources. We also collected information on whether a chemical has been ‘monitored but never detected’ or ‘monitored and detected’. If it was stated in the biomonitoring programs that the analyte was a metabolite of a specific parent compound, we paired the metabolite and the parent compound for comparison with the known FCCs. For example, the analyte mono-ethyl phthalate (CAS 863029-89-4) is listed as a metabolite of di-ethyl phthalate (CAS 84-66-2) in NHANES, and we used both CAS Registry Numbers in the comparisons to the known FCCs. In this way, we ensured that FCCs were identified in the biomonitoring programs regardless of whether detection in human samples was reported for parent compounds or their metabolites. We manually added CAS Registry Numbers to chemicals missing these identifiers in the biomonitoring lists to enable their comparisons to the FCCs.

In step 1B, the data set ‘biomarkers’ was downloaded from the Exposome Explorer, and the full content of the Blood Exposome Database was retrieved. From the HMDB, all chemicals were included that were labeled by metabolite status as ‘detected and quantified’, ‘detected but not quantified’, and ‘expected but not quantified’. The metabolome/exposome databases do not systematically report links between parent compounds and metabolites. We used these chemical lists from the metabolome/exposome databases without any further editing.

Based on their CAS Registry Numbers, InChI Keys, or SMILES identifiers, FCCs were then compared to the chemical lists retrieved from the biomonitoring programs and metabolome/exposome databases. These comparisons were performed by means of Python (v3.10.8) pandas package (v1.5.3).

### Systematic evidence mapping (step 2)

#### Prioritization and grouping of FCCs

In step 2, we focused on the FCCs that were not found in any of the sources of step 1, i.e., all FCCs, or their metabolites, that have never been included in a biomonitoring program (regardless of whether they have been detected or not) and all FCCs that did not generate any match in the metabolome/exposome databases. These FCCs not monitored in any of the sources of step 1 were candidates for the systematic evidence mapping in step 2. For this step, we prioritized FCCs that have at least five database entries in the FCCmigex, reporting their detection in migrates and/or extracts of FCMs. To verify the absence of any prioritized chemicals in step 1, we also searched the HMDB for the chemical names that are used in the FCCmigex database and in Norman SLE.

For further data analysis and interpretation, prioritized FCCs were assigned to chemical groups based on functional categories and/or chemical structures. During grouping, we referred to the primary literature included in this systematic evidence map and in the FCCmigex database to understand the function and/or chemical features of an FCC. Additionally, we used the tool Classyfire [45], the Plastics Additives Handbook [46], and expert knowledge to group FCCs based on their applications in FCMs and/or chemical features, such as functional groups and structural properties.

#### Literature searches and screening

For each of the prioritized FCCs, individual literature searches were performed. For PubMed, WoS, and ScienceDirect, search strategies included the chemical name as used in the FCCdb or the FCCmigex, and generic search terms related to human biomonitoring (e.g., human, blood, urine, biomonitoring) that were connected by the Boolean operator OR. Searches in CAS SciFinder^n^ used CAS Registry Numbers instead of chemical names. Search strings and settings were slightly adapted depending on the requirements of each database. The searches were not restricted by publication date or language and included all literature published by February 2023. Full details on search strings, applied filters, and settings have been published previously [29].

Individual literature searches were stored in separate Endnote files, from which duplicates were removed. All individual libraries were uploaded into the online evidence synthesis tool Cadima [47], where further duplicates were deleted. The references were then screened in a two-level process, beginning with title-and-abstract screening and followed by full-text screening. During the screening, the eligibility criteria specified in the protocol were applied to all prioritized FCCs that were analyzed in the respective reference [29]. In brief, studies were considered eligible and included in the systematic evidence map if the analyzed sample originated from a human specimen (e.g., urine, blood, and breast milk) and at least one prioritized FCC was analyzed. Ten percent of the references were independently screened by two reviewers in parallel at title-and-abstract and full-text levels, and disagreements were resolved bilaterally. Reasons for exclusion were recorded during full-text screening.

#### Data extraction

Eligible studies were used to collect information on whether FCCs have been monitored in human samples and if they have been detected. Details on the sample type and analytical approaches were part of the data extraction process (see Supplementary Information). The process was based on the data extraction software tool SciExtract [5] which allowed us to use precoded options to systematically compile the data. SciExtract was also used to organize and manage the workflow and to store the extracted data.

### Hazard mapping

For FCCs included in the biomonitoring programs (step 1A) and those prioritized in step 2, we compiled the hazard properties according to human-health-related criteria described in the EU’s Chemicals Strategy for Sustainability (CSS) [48]. The CSS seeks to ban the most harmful chemicals from consumer products, including FCMs, and defines chemicals as ‘most harmful’ to human health if they are carcinogenic, mutagenic or toxic to reproduction (CMR) or exhibit specific target organ toxicity (STOT). Hazards associated with endocrine-disrupting properties, persistence, bioaccumulation, and mobility of a chemical are also mentioned in the CSS but were not included in this analysis. We consulted the European Chemicals Agency’s (ECHA) Classification and Labelling Inventory aligned with the Globally Harmonized System (GHS) for chemical classification and labeling [49] and referred to GHS-aligned classifications by the Japanese Government [50] for identifying human health-related hazards. Following the GHS criteria for classification and labeling, we identified chemicals as ‘high concern’ if they exhibit CMR properties belonging to categories 1A and 1B (known and presumed CMR, respectively) and/or have been classified as STOT category 1 after repeated exposure (RE) (Fig. S1). Chemicals of ‘medium concern’ were those suspected to have CMR and/or STOT RE properties, as indicated by their classifications in category 2. Chemicals that have been classified based on other concerns, such as aquatic toxicity or skin sensitization, were marked as ‘other concern’. FCCs with data in at least one hazard category and without any classification were labeled as ‘not classified’. FCCs that were not included in the hazard inventories, or for which no data were available in any hazard category, were labelled with ‘no hazard data’.

## Results

### Overall evidence for the presence of FCCs in humans

**Fig. 2:**
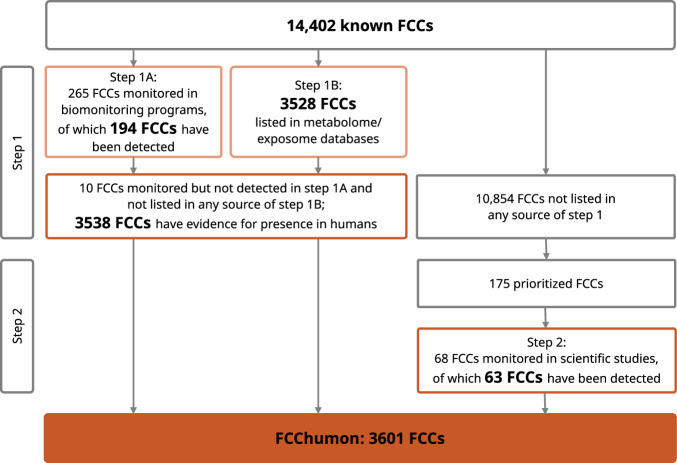
FCCs with evidence for presence in humans that form the FCChumon database. Schematic representation of the FCCs monitored and detected in biomonitoring programs and/or listed in metabolome/exposome databases (step 1) and additional FCCs detected in humans, based on evidence from the scientific literature for a set of prioritized FCCs (step 2).

For a total of 3601 (or 25%) of the 14,402 known FCCs, we found evidence for their presence in human samples (Fig. 2). Of these, 194 FCCs have been detected in biomonitoring programs, and 3528 FCCs are listed in metabolome/exposome databases, with an overlap of 184 FCCs found in both types of sources. The total of 3601 FCCs also includes 63 out of 175 prioritized FCCs that have been detected in humans according to the results of the systematic evidence map (step 2).

Based on the results of this stepwise approach, we set up the FCChumon database, which is provided as an interactive tool that is freely available, searchable, and linking to the relevant sources (https://www.foodpackagingforum.org/fcchumon).

### Analysis of biomonitoring programs and metabolome/exposome databases

**Fig. 3:**
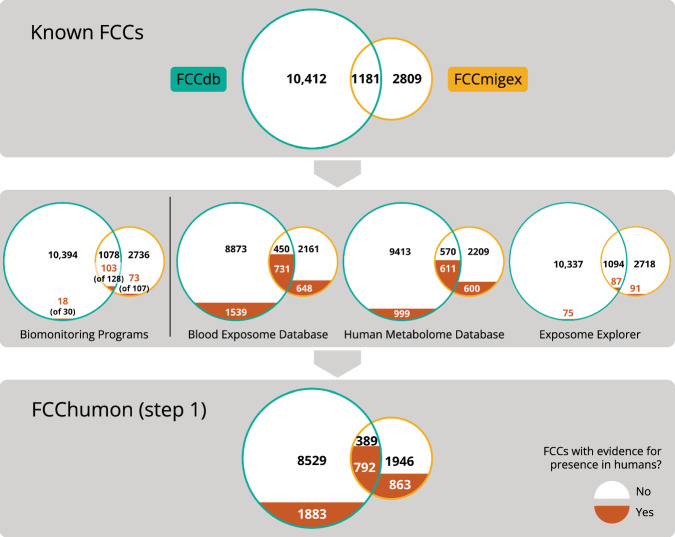
Overview of FCCs with evidence for presence in human samples. The upper panel illustrates the FCCs from the FCCdb (green outline), the FCCmigex (yellow outline), and their overlap. The left part of the middle panel shows the number of known FCCs that have been detected in biomonitoring programs and, in brackets, the total number of monitored FCCs. The right part of the middle panel displays the FCCs that are listed in metabolome/exposome databases. FCCs that have been detected in humans are indicated by the orange filling of the respective areas; white areas represent FCCs without any evidence of the presence in humans and the FCCs that have been monitored but not detected. The figure in the lower panel is the result of the overall comparison of the known FCCs with all sources of step 1.

In step 1, we identified 3538 FCCs that have been detected in humans, which can be divided into 1883, 863, and 792 FCCs that are included only in the FCCdb, only in the FCCmigex, and in both databases, respectively (Fig. 3, lower panel). These numbers indicate that 23% of the FCCs in the FCCdb and 41% of the FCCs in the FCCmigex are listed in at least one of the sources in Step 1. Sixty-seven percent of FCCs that are listed in both FCC databases have evidence of presence in humans.

Of the 265 FCCs monitored in at least one of the five biomonitoring programs, 194 FCCs (or their metabolites) have been detected in human samples, and 71 FCCs (or their metabolites) have been monitored but not detected in any of the biomonitoring programs (Fig. 3, middle panel; Table S1). The most extensive national program, NHANES, has monitored over 400 different chemicals in human samples since 1999, and 154 of these are FCCs (Figure S2). We also found 84, 66, 66, and 25 FCCs with evidence for the presence in humans in the biomonitoring programs CHMS, HBM4EU, Biomonitoring California, and KoNEHS, respectively. One hundred and twenty-four FCCs have only been monitored in a single biomonitoring program, and 55 of these have not been detected, whereas 13 FCCs have been included across all five programs, of which 8 have been detected in all programs (Figure S3; Table S1).

The overlap of known FCCs with metabolome/exposome databases is much larger than the overlap with biomonitoring programs: of the three metabolome/exposome databases, the Blood Exposome Database includes the highest number of FCCs (2918 FCCs), followed by the HMDB (2211 FCCs) and the Exposome Explorer (253 FCCs) (Fig. 3, middle panel; Figure S4). The HMDB lists 367, 1072, and 772 FCCs that are labelled as “detected and quantified”, “detected but not quantified”, and “expected but not quantified”, respectively, according to the classification system of the database (Figure S5) [36].

Sixty-one out of the 71 FCCs that have been monitored but not detected in biomonitoring programs are listed in at least one of the metabolome/exposome databases. This means that only 10 FCCs fall under the category “monitored but not detected” in step 1 (Fig. 2).

### Systematic evidence mapping of prioritized FCCs

In step 1 we show that 75% of the known FCCs are not listed in any of the biomonitoring programs or metabolome/exposome databases. However, for some of these FCCs, scoping searches resulted in additional evidence from the primary literature. Therefore, we decided to systematically map the evidence for 175 FCCs which we prioritized based on the number of FCCmigex database entries that report their detection in FCMs.

In this systematic approach, we found 3152 scientific studies for 147 out of the 175 prioritized FCCs (Figure S6) and considered 251 and 159 studies eligible after title-and abstract and full-text screening, respectively. These studies refer to 68 FCCs – for the other 107 FCCs, no studies fulfilled the eligibility criteria.

**Fig. 4:**
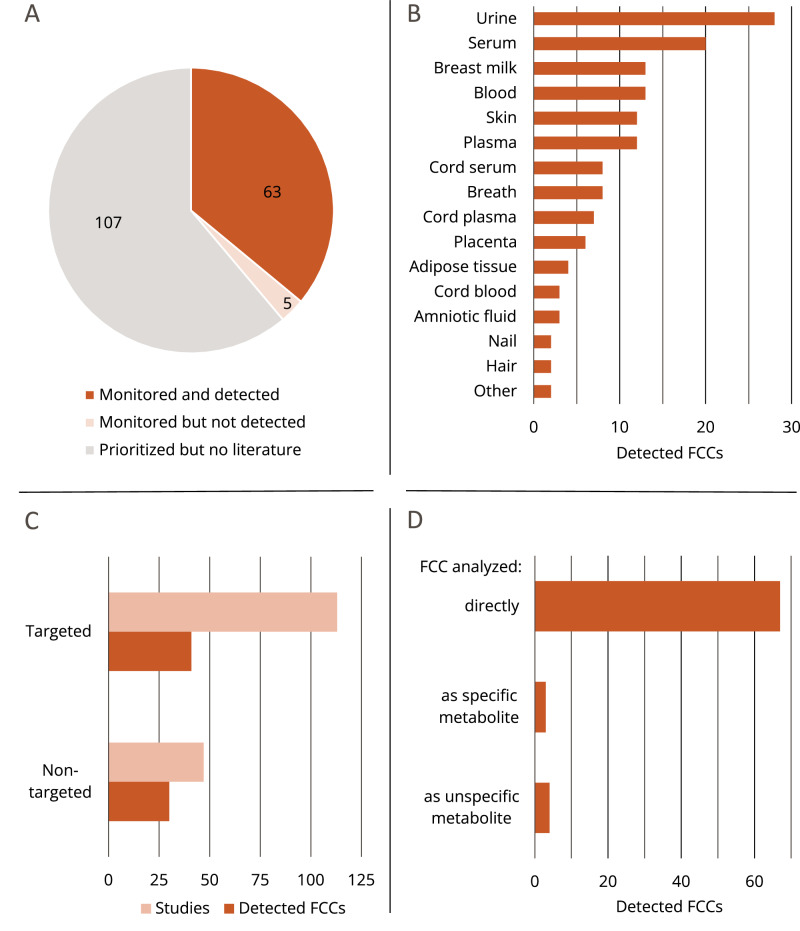
Results of the systematic evidence map addressing the presence of 175 prioritized FCCs in humans (step 2). **A** Numbers of FCCs with and without evidence from the primary scientific literature indicating their presence in humans. **B** Types of human samples in which the 63 FCCs have been detected (multiple sample types possible). **C** Types of applied analytical methods per study and per detected FCC. **D** Numbers of FCCs that have been analyzed directly (as parent compound) or as specific or unspecific metabolite.

Of the 68 FCCs for which scientific studies were found, 63 have been detected in human samples and five have been monitored, but not detected, i.e., Irganox 1330 (CAS 1709-70-2), 2,6-(1,1-dimethylethyl)phenol (CAS 128-39-2), phenyl-bis-(2,4,6-trimethylbenzoyl) phosphinoxid (CAS 162881-26-7), 2,5-bis(5-tert-butyl-2-benzoxazolyl) thiophene (CAS 7128-64-5), and Tinuvin 622 (CAS 65447-77-0) (Fig. 4A). The detected chemicals have been detected in urine (28 FCCs), serum (20), blood (13), and plasma (12) (Fig. 4B). FCCs have also been found in breast milk (13) and samples taken from umbilical cords (18) and placentas (6). One hundred and thirteen studies have used targeted analyses, whereas 47 studies have used non-targeted approaches (Fig. 4C), and only one study has applied both methods [51]. The vast majority of FCCs have been detected directly, i.e. as parent compounds, in human samples (Fig. 4D), while antioxidant 1098 (CAS 23128-74-7) and Irganox 1035 (CAS 4148-35-9) have been putatively identified based on an unspecific common metabolite in one study [52].

### FCCs monitored in humans

#### FCCs detected in biomonitoring programs

**Fig. 5:**
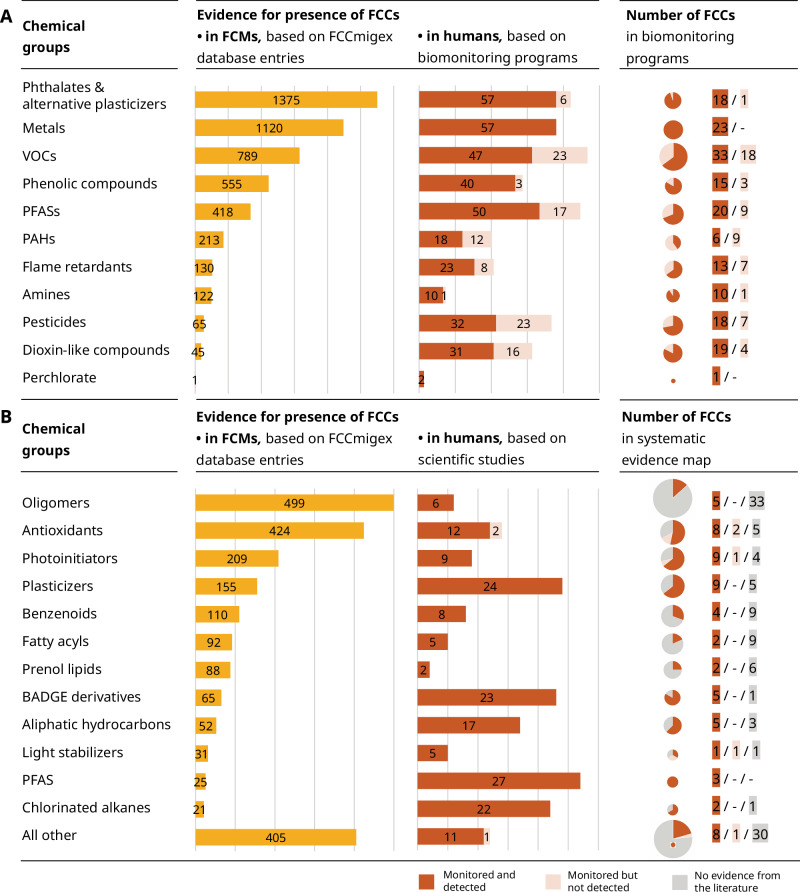
Evidence for presence of FCC groups in FCMs and in humans. **A** 235 FCCs detected in FCMs and included in biomonitoring programs (step 1A). **B** 175 FCCs prioritized based on their detection in FCMs and their absence in step 1 (step 2). The yellow bar charts illustrate the evidence for the presence of FCC groups in FCMs, based on the sum of database entries from the FCCmigex that report the detection of FCCs in FCMs. The orange bar charts show the evidence of the presence of FCC groups in humans. In step 1A, this is based on the number of biomonitoring programs that have monitored individual FCCs in humans and the addition of these counts by group. In step 2, the orange bars represent the number of studies that have monitored at least one FCC of the respective group. The pie charts show how many FCCs per group have been monitored and detected at least once and how many FCCs have been monitored but not detected in any sample. For step 2, the pie charts also include the chemicals for which there is no evidence in the scientific literature.

Among the 235 FCCs present in FCMs that have been included in human biomonitoring programs, there are 51 volatile organic compounds (VOCs), 29 per- and polyfluoroalkyl substances (PFAS), 25 pesticides, 23 metals, 23 dioxin-like compounds, 20 flame retardants, and 19 phthalates and their alternatives (Fig. 5A, right panel; Table S1). Phthalates and alternative plasticizers, and metals are frequently detected FCCs in FCMs and have also been often found in humans (Fig. 5A, bar charts). Furthermore, PFAS, VOCs, and phenolic compounds, including bisphenols, parabens, and benzophenones, have been frequently monitored and detected in FCMs and in humans. In contrast, for dioxin-like compounds, pesticides, flame retardants, polyaromatic hydrocarbons (PAHs), amines, and perchlorate there is less evidence for their presence in FCMs. Interestingly, 71 of the 95 FCCs belonging to these six groups would not be expected to be present in FCMs, since they are not included in the FCCdb (Table S1). The evidence for presence of FCCs in FCMs varies widely between but also within chemical groups. For example, the VOC styrene (CAS 100-42-5) has been listed 99 times as “detected in FCMs” in the FCCmigex database, while 16 other VOCs found in humans have been listed less than ten times each (Table S1). The presence of styrene, or its metabolites, in humans has been shown by NHANES, CHMS, and KoNEHS, but there is no evidence for 18 of the 51 VOCs from any of the five biomonitoring programs.

#### FCCs included in the systematic evidence map

Among the 175 FCCs included in the systematic evidence map, there are 38 oligomers (mainly siloxane, polyamide, and polyethylene terephthalate (PET) derivatives), 15 antioxidants and degradation products, 14 photoinitiators, and 14 plasticizers (Fig. 5B, right panel; Table S2).

For oligomers and antioxidants and their degradation products, 424 and 499 FCCmigex database entries, respectively, imply that FCMs play a role in human exposure to these chemical groups (Fig. 5B). However, there is limited evidence for the presence of antioxidants and oligomers in humans, as indicated by 6 and 12 studies, respectively, reporting the detection of the chemicals of these groups. For only five out of 38 prioritized oligomers, we found evidence for their detection in humans: a PET cyclic trimer (CAS 7441-32-9), three cyclic siloxanes (D7, CAS 107-50-6; D8, CAS 556-68-3 and D9, CAS 556-71-8), and 1,6-dioxacyclododecane-7,12-dione (CAS 777-95-7) (Table S2). With 209 FCCmigex database entries and 9 studies reporting detection in humans, photoinitiators are regularly found in FCMs, but less frequently monitored in humans. For the five BADGE derivatives BADGE·H_2_O, BADGE·2H_2_O, BADGE·HCl, BADGE·2HCl, and BADGE·H_2_O·HCl, 23 studies confirm the detection of at least one of these FCCs in humans. In addition, they have 65 database entries in the FCCmigex, confirming their regular detection in migrates and/or extracts from coated metal FCMs.

### FCCs of concern

**Fig. 6:**
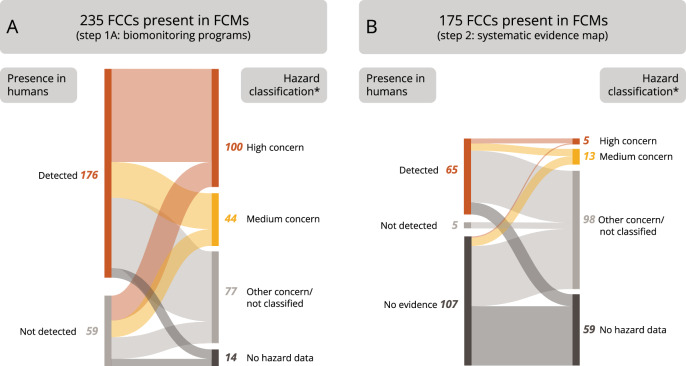
Relationships between hazard classifications of FCCs and evidence for their presence in humans. **A** 235 FCCs detected in FCMs and included in biomonitoring programs. **B** 175 FCCs prioritized based on their detection in FCMs and their absence in step 1. On the left side of both Sankey diagrams, the number of FCCs monitored and detected in humans (red), monitored but not detected in humans (light gray), and without any evidence for the presence in humans (dark gray) are shown. On the right sides, the diagrams visualize the number of chemicals of high (red) and medium concern (yellow), chemicals of other concerns or not classified chemicals (light gray), and chemicals with no hazard data (dark gray). The thickness of connecting lines represents the numbers of chemicals that belong to a hazard category and their evidence for presence in humans. *Many hazard classifications lack information for specific hazard categories. This means that chemicals may be newly categorized or reassigned to other hazard categories when more information becomes available in the future.

Of the 235 FCCs included in biomonitoring studies and with evidence for their presence in FCMs, 100 FCCs have hazard properties of high concern for human health, and 44 FCCs have hazard properties of medium concern, i.e., they are assigned to categories 1 and 2, respectively (Fig. 6A, Table S1). Among the FCCs detected in humans are several category 1 A and 1B carcinogens, of which, e.g., styrene, benzophenone (CAS 119-61-9), formaldehyde (CAS 50-00-0), and cadmium (CAS 7440-43-9) have also been frequently found in FCMs. Dozens of FCCs are classified as toxic to reproduction, for example, nine phthalates, which are all classified as 1B reprotoxicants. Over 30 FCCs are mutagens (e.g., benzene (CAS 71-43-2), lead, cadmium, and cobalt), and many more exhibit specific target organ toxicity after repeated exposure (e.g., 4,4’-methylenedianiline (CAS 101-77-9) and perfluorooctanoic acid (CAS 335-67-1)). Seventy-seven FCCs have other concerns or have not been classified as hazardous based on the available data, and 14 do not have hazard data or are not listed.

Among the 175 FCCs included in the systematic evidence map, 5 and 13 FCCs are classified in categories 1 and 2, respectively, resulting in high and medium concern for CMR and/or STOT RE properties (Fig. 6B, Table S2). Di-n-octylisophthalate (CAS 137-89-3), 2-benzyl-2-(dimethylamino)-4-morpholino-butyrophenone (CAS 119313-12-1), ethyl-4-dimethylaminobenzoate (CAS 10287-53-3), and medium-chain chlorinated paraffins (CAS 85535-85-9) are reproductive toxicants of high concern (category 1B) and have been detected in FCMs and in humans. For the category 1B carcinogen 2,4’-methylenedianiline (CAS 1208-52-2), however, we found no evidence concerning its presence in humans. Ninety-eight FCCs are allocated to other hazard categories or have not been classified, and 59 FCCs are not listed in the hazard inventories, indicating a lack of data for these chemicals. Based on this evidence map, 49 FCCs without hazard data have also never been targeted in human samples, but they are known to migrate so the implications of the probable human exposure from these FCCs are unknown. Among these are 29 oligomers that have been mainly detected in PA, PET, and siloxane FCMs.

## Discussion

### Relevance of this study

There is evidence of human exposure for at least 3601 (or 25%) of the known FCCs (Fig. 1). While other exposure sources (than FCMs) exist for FCCs, it is likely that humans are exposed to more FCCs than reported here, as we only searched the scientific literature for a small subset of chemicals. The novel database on FCCs monitored in humans (FCChumon) lends itself to integration with our previously published database of chemicals present in/migrating from specific FCMs (FCCmigex) [5], thereby enabling hypothesis-driven research for closing pertinent knowledge gaps on human exposure to chemicals originating from FCMs. Together, these databases can also be used as information sources for elucidating FCCs’ health impacts and highlighting other priority research needs.

### Parent compounds vs. metabolites

For the exposure assessment of chemicals with well-known metabolic fate in humans, such as phthalates and certain VOCs, metabolites instead of their parent compounds are monitored [53, 54]. We considered this aspect when comparing FCCs to chemicals from the biomonitoring programs and when analyzing the primary literature. Various tools could support identifying FCC metabolites by predicting chemical biotransformation [55, 56], but they are associated with large scientific uncertainty, as shown, e.g., for the metabolism of agrochemicals in rats [57] or for 15 structurally different groups of flame retardants [58]. Given the high number of FCCs included in this study, we did not attempt to systematically predict potential metabolites and only considered information on specific metabolites if it was readily available in the biomonitoring programs. Only one unspecific metabolite was identified in the systematic evidence map, indicating potential exposure to two antioxidants [52].

### Focus on chemical groups

FCMs are a well-known and relevant exposure source for phthalates and their alternatives, metals, VOCs, and phenolic compounds. These chemicals are regularly monitored and detected in human biomonitoring programs and frequently found in FCMs (Fig. 5A), and there is ample evidence for their migration, e.g. [17, 59–61]. There is also evidence for the presence of PFAS in humans and in FCMs. Although most PFAS have never been authorized for food contact use [62], the contribution of food packaging to human exposure has been mapped [63]. Dioxin-like compounds, many pesticides, and flame retardants are not intentionally added FCCs, but they may be present in FCMs because they are introduced or formed during FCM use, manufacture, and recycling, as their detection in FCMs shows [64–66]. FCMs may therefore contribute to human exposure to FCCs intentionally used in the manufacture of FCMs, various types of NIAS, and illicitly added chemicals. Yet, for most FCCs, comprehensive assessments of the relative contribution of FCMs to human body burden are missing.

Antioxidants are of special interest because many are high-production volume chemicals that are widely used in plastic food packaging [67] and robust evidence for their presence in FCMs exists (Fig. 5B, Table S2). Important groups of antioxidants are sterically hindered phenols and phosphite antioxidants that are very common in FCMs, e.g., Irgafos 168 (CAS 31570-04-4), Irganox 1076 (CAS 2082-79-3), and Irganox 1010 (CAS 6683-19-8). However, neither of these substances is included in the biomonitoring programs and exposome/metabolome databases (step 1), and we found only limited evidence for their presence in humans in step 2 [52, 68, 69]. Major degradation products of these antioxidants, such as 2,4-di-tert-butylphenol (CAS 96-76-4), 2,6-di-tert-butylbenzoquinone (CAS 719-22-2), and tris(2,4-di-tert-butylphenyl)phosphate (CAS 95906-11-9), have been detected in humans in a few studies, but at high levels and with frequent detection in sampled populations [70–72]. These results show that the contribution of FCMs to human exposure to antioxidants and their degradation products has not yet received much attention. Such gaps need to be filled by better understanding the overall exposure to antioxidants and their metabolism in humans.

Oligomers are another group of FCCs requiring more attention. PET, PA, and siloxane oligomers are known side-products of polymerization, and they have been detected in extracts and migrates of FCMs. There is however only very limited evidence for their presence in humans, e.g. for PET oligomers [73]. This is likely due to the challenging chemical analysis of oligomers, especially in complex media, such as human samples, and the fact that chemical standards required for the identification and quantification of oligomers are rarely available [74, 75]. BADGE and its derivatives are commonly observed side-products formed during the polymerization of epoxy resins [76]. Toxic effects, such as endocrine disruption, genotoxicity, and allergic reactions, have been linked to BADGE derivatives and epoxy resins, but information on their toxicity is still limited [77]. Seventeen BADGE derivatives have been detected in extracts or migrates of FCMs, and five of them have been found in humans. This illustrates that targeted analysis of structurally related chemicals is possible and should be prioritized, to close this important knowledge gap on human exposure to expected side-products of polymerization reactions [76].

Photoinitiators form a group of structurally diverse FCCs that are used in various FCMs, such as coatings, printing inks, and adhesives [78]. While there is substantial evidence for their presence in FCMs, their presence in human samples has not been extensively investigated. Liu and Mabury showed that 18 photoinitiators and their sulfoxidation products are present in human sera [79], and human exposure, environmental occurrence, and toxicity of 25 photoinitiators have recently been reviewed [78]. According to the FCCmigex and FCChumon databases, several of these photoinitiators have been detected in FCMs and there is evidence for human exposure. Among these, benzophenone (CAS 119-61-9) is the most frequently detected photoinitiator in FCMs. Since benzophenone is a presumed carcinogen (class 1B, Table S1) as well as a suspected endocrine disruptor [80], exposure via FCMs should be prevented.

### Limitations affecting data interpretation

The sources used for the compilation of the FCChumon data vary with respect to the chemical space, curation level, and details provided. In general, we consider data collected in biomonitoring programs (Step 1A) as having a high level of confidence because they are usually derived from a representative population by following strict analytical standards and guidelines [81]. However, only a limited number of several hundred chemicals is monitored in these programs. We also rate the results of step 2 with a high level of confidence because they were generated by the robust approach of a systematic evidence map (including data extraction by a trained team of scientists but excluding the quality rating of each included study [29]). Conversely, the metabolome/exposome databases contain many thousands of different chemicals that have been assembled by different means, also including automated approaches [36, 38]. The matches between the known FCCs and these databases may therefore require further review before being used in future assessments (e.g., by checking the “metabolite status” integrated in the HMDB).

Some of the FCCs listed in the FCCdb and FCCmigex consist of chemical mixtures of, e.g., polymeric molecules, stereoisomers, or structural isomers. Converting the CAS Registry Number of such mixtures into other identifiers was not always possible and could therefore result in some FCCs not being found in some sources of step 1. For example, short-chain chlorinated paraffins (SCCPs, CAS 85535-84-8) and medium-chain chlorinated paraffins (MCCPs, CAS 85535-85-9) do not have any identifiers other than CAS and were not matched in step 1, but we found ample evidence for the presence of these mixtures in humans in step 2, because they have been monitored regularly and the chemical names are reported in a standardized manner in the primary literature e.g. [82–84]. Nonylphenol (CAS 25154-52-3) is another example of a mixture of undefined stereoisomers and structural isomers that was not found in step 1 but prioritized in step 2. However, due to the listing of more defined nonylphenol isomers in the FCC databases as well as the metabolome/exposome databases, we decided to exclude this technical mixture from the systematic evidence map. These examples show that searches for (alternative) names and/or identifiers were helpful during the systematic evidence map and may be recommended for users of the FCChumon database.

### Implications for assessing and managing FCCs

The data presented here lend support to the possible contribution of FCMs towards human exposure to FCCs. Since there are various FCCs with hazard properties of concern among the chemicals detected in humans and FCMs, their use in FCMs should be restricted to minimize human exposure. This is now recognized and currently under discussion for a few of these chemicals, including PFAS [85, 86], BPA [10, 16] and phthalates [87]. However, it does not mean that the remaining FCCs can be considered safe, as shown, e.g., by the absence of biomonitoring and hazard data for 107 (61%) and 59 (34%), respectively, of the 175 FCCs included in step 2. Importantly, even for chemicals where hazard data have been submitted to authorities there are significant data gaps for one or more hazard categories, as has been demonstrated for certain PFAS [62, 88]. For FCCs migrating into foods, such related hazard data gaps need to be filled with high priority to characterize risk on human health [89]. This is especially urgent for intentionally added FCCs found at high levels in humans, such as antioxidants and photoinitiators, and expected NIAS, such as oligomers and BADGE derivatives.

In summary, this study systematically maps 3601 chemicals from different FCAs (food packaging, tableware, etc.) for which there is evidence for human exposure, and for 10,786 FCCs, no evidence could be provided at all. Only 15 FCCs have been monitored but have never been detected in humans. Based on two subsets totalling 410 FCCs, this study further identifies 105 FCCs of high concern due to their hazard properties and highlights the many data gaps related to hazards and human health risks. We make these data accessible in the user-friendly, freely accessible FCChumon dashboard, which complements our previously published FCCmigex dashboard on extractable and migrating FCCs. In combination, FCChumon and FCCmigex enable the prioritization of FCCs requiring more detailed investigations, either because they are frequently found in FCMs, despite having only little or no information on their presence in humans, or because they are measured in humans but lack hazard information. Furthermore, this evidence base supports policy and decision-making and highlights the urgent need to ban the most hazardous chemicals shown to migrate from food packaging and other types of FCAs into foods, to protect human health.

## Supplementary information

Protocol for assessing the evidence of food contact chemicals monitored in humans

Supplementary information

Supplementary Tables

## Data Availability

The data are publicly and freely available as interactive dashboard that is based on Microsoft PowerBI under the following link (https://www.foodpackagingforum.org/fcchumon). The references that were included in the systematic evidence map (step 2) are also provided under this link.
