# Supplementary material for: Evidence for widespread human exposure to food contact chemicals
Source: J Expo Sci Environ Epidemiol. 2024 Sep 17;35(3):330–41. doi: 10.1038/s41370-024-00718-2 (PMC12069106; doi:10.1038/s41370-024-00718-2)
Supplement: Supplementary file 1 — Protocol for assessing the evidence of food contact chemicals monitored in humans [file 41370_2024_718_MOESM1_ESM.pdf]

Protocol for assessing the evidence of food contact chemicals monitored in humans

Registration of version 2.0:
Protocol update uploaded to Zenodo and attributed the following digital object identifier (DOI) 10.5281/zenodo.7857837 (April 24, 2023).

Authors:
Birgit Geueke[1*], Lindsey V. Parkinson[1], Jozica Dolenc[2], Ksenia J. Groh[3], Christopher D. Kassotis[4], Maricel V. Maffini[5], Olwenn V. Martin[6], Lisa Zimmermann[1], Jane Muncke[1]

*corresponding author: birgit.geueke@fp-forum.org

[1]Food Packaging Forum Foundation, Zurich, Switzerland

[2]Chemistry | Biology | Pharmacy Information Center, ETH Zurich, Zurich, Switzerland

[3]Eawag, Swiss Federal Institute of Aquatic Science and Technology, Dübendorf, Switzerland

[4]Institute of Environmental Health Sciences and Department of Pharmacology, Wayne State University, Detroit, MI, USA

[5]Independent consultant, Frederick, MD, USA

[6]Department of Arts & Science, University College, London, UK

Table of contents

1 Introduction 3
2 Objectives..4
3 Planning...5
3.1 Definition of key terms 5
3.2 Authors' contributions and scientific advisory group 6
3.3 Research question 7
3.4 Scoping exercise 7
3.5 Consultation process 8
4 Information sources..11
4.1 Biomonitoring programs...11
4.2 Metabolome/exposome databases 12
4.3 Bibliographic databases 12
5 Methods 13
5.1 Tier 1: Comparing the Universe of known FCCs with tertiary sources 13
5.1.1 Biomonitoring programs 13
5.1.2 Metabolome and exposome databases.......................................14
5.2 Tier 2: Mapping further evidence for targeted FCCs 14
5.2.1 Prioritization of FCCs 14
5.2.2 Eligibility criteria..15
5.2.3 Search strategies 17
5.2.4 Management and screening of the literature.............................19
5.2.5 Data coding and extraction 21
5.2.6 Study quality assessment 23
5.3 Integration of results and reporting 23
6 Financial Support..25
7 Declaration of Competing Financial Interests 25
8 References 26

1 Introduction

Foods and beverages come into contact with food packaging and other food contact articles (FCAs) during their production, transport, processing, preparation, and consumption. FCAs consist of a wide variety of materials (food contact materials, FCMs) that often release chemicals (food contact chemicals, FCCs) into the food/beverage. This process is called chemical migration and leads to continuous human exposure to FCCs. The human health effects related to such chronic, low-level chemical exposures to FCCs are poorly understood (Muncke et al., 2020). The available scientific evidence mostly focuses on a few well studied FCCs that are known chemicals of concern such as bisphenol A and ortho-phthalates (Warner & Flaws, 2018).

To better address these issues, the Food Packaging Forum and partners initiated the Food Contact Chemicals and Human Health (FCCH) project, where we systematically investigate the wide variety of FCCs and their potential impact on human health in several stages.

First, we compiled the Food Contact Chemicals Database (FCCdb), which is an inventory of 12,285 FCCs known to be intentionally added or associated with the manufacture of FCMs (Groh et al., 2021). The FCCdb is based on 67 FCC lists from publicly available sources, such as regulatory lists and industry inventories. However, an FCC being listed in the FCCdb is not necessarily used in the manufacture of FCMs. The database does not systematically capture non-intentionally added substances (NIAS) that may also be present in FCMs and FCAs. NIAS are not added on purpose during the production of FCMs, but they may nevertheless migrate into foods and beverages from the final FCA. Typical examples of NIAS are reaction side products, breakdown products, and contaminants (Geueke, 2018; Nerín et al., 2022).

Second, we published the Database on Migrating and Extractable Food Contact Chemicals (FCCmigex) (Geueke et al., 2022). This systematic evidence map collates empirical data on FCCs that have been measured in migrates and extracts of all types of FCMs and FCAs. At the time of its publication, the FCCmigex contained 2881 FCCs that have been detected in six different FCM groups (plastics, paper & board, metals, multi-materials, glass & ceramic, and other FCMs).

Together, these two databases contain a total of 14,153 FCCs, which we call the Universe of known FCCs (Geueke et al., 2022; Groh et al., 2021) (Figure 1). 1013 FCCs appear in both datasets, whereas 11,272 FCCs are included in the FCCdb only. In contrast, 1868 FCCs have been detected in migrates and/or extracts of FCMs, but are not listed in the FCCdb. This observation implies that these FCCs are either NIAS or were intentionally used without being included in any of the 67 lists that were used to compile the FCCdb.

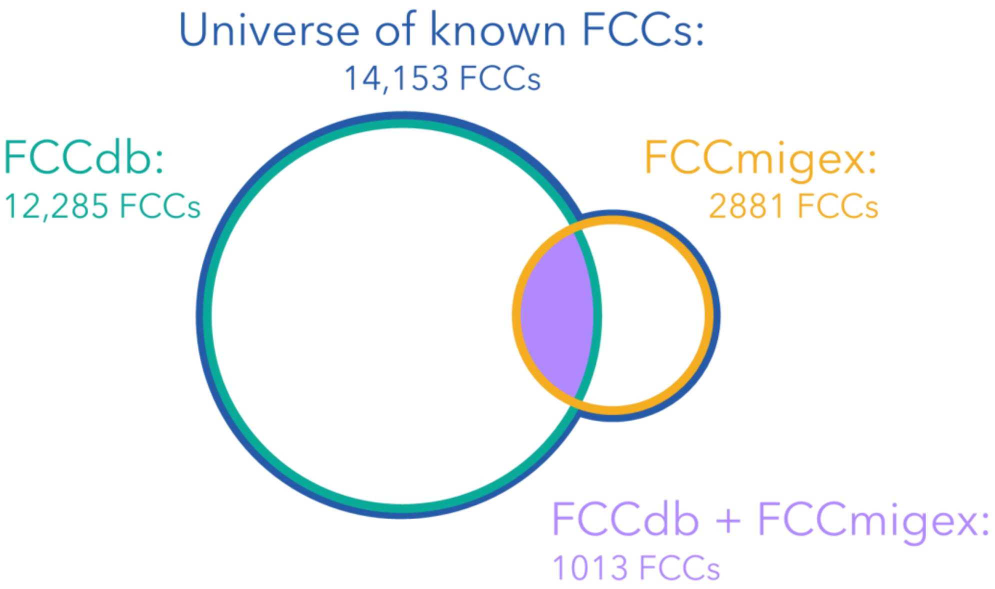

Figure 1. Universe of known FCCs (blue outline). Schematic overview of the intentionally used FCCs (green circle) and the FCCs with evidence of migration/extraction (yellow circle). The chemicals are either part of the FCCdb, FCCmigex, or both databases (indicated by the purple overlap) (Geueke et al., 2022; Groh et al., 2021).

In the next stage of this project to which this protocol applies, we will collect evidence for FCCs monitored in human samples as this indicates human exposure. This question has not been addressed systematically so far. Together with the information provided in the FCCdb and the FCCmigex, we will then be able to further categorize FCCs based on their potential uses and available hazard data, their evidence for originating from FCMs/FCAs, and their occurrence in the human body. In addition, FCCs for which no human biomonitoring data were found will be identified.

2 Objectives

The overall objective of the evidence map described here is to systematically document the available scientific evidence for known FCCs that have been monitored in human samples. More specifically, we will:

- Identify FCCs that have been monitored in the human body by (i) comparing biomonitoring programs and metabolome and exposome databases to the Universe of known FCCs as well as (ii) identifying primary scientific literature containing additional evidence for some of the FCCs not included in the sources used in (i).
- Present the data in a user-friendly and understandable way and provide the bibliographic information on the underlying references.
- Identify data gaps and research needs, discuss limitations and uncertainties, and publish these considerations and the key findings in a summary article.

3 Planning

3.1 Definition of key terms

The following list provides our working definitions for important terms used in this protocol. Alternative definitions may exist in the scientific literature. This list is sorted by topic, not alphabetically.

Food contact article (FCA): A product or item which intentionally comes into contact with food, such as storage containers, conveyor belts, tubes, processing equipment, packaging, tableware or cooking utensils.

Food contact material (FCM): Any type of material that is used in the manufacture of FCAs. Typically, FCMs are in direct contact with food, but materials that are not in direct contact can also be a source of chemical migration and can be considered FCMs (e.g., printing inks, adhesives).

Food contact chemical (FCC): Chemical substances used in the manufacture of FCMs and FCAs and/or present in the final FCMs and FCAs. FCCs include the intentionally used starting substances, substances generated during manufacture of an FCM/FCA and non-intentionally added substances (NIAS).

Non-intentionally added substances (NIAS): NIAS comprise all substances that have not been added for a technical reason during manufacturing of FCMs, but that are nevertheless present in the final FCM or FCA.

Food Contact Chemicals Database (FCCdb): The database was compiled from 67 lists of FCCs from publicly available sources, including regulatory lists and industry inventories. It contains 12,285 distinct FCCs that may be used intentionally in or are associated with the manufacture of FCMs (Groh et al., 2021).

Database on migrating and extractable food contact chemicals (FCCmigex): The database was compiled from primary literature and contains more than 3000 FCCs that have been measured in extracts and/or migrates of FCMs and FCAs (Geueke et al., 2022).

Database on food contact chemicals monitored in humans (FCChumon): The database will be the product of the planned evidence map as it is described in this protocol. It will provide evidence for FCCs that have been monitored in human samples.

Universe of known FCCs: The sum of all FCCs that have been listed in the FCCdb and FCCmigex (Geueke et al., 2022; Groh et al., 2021). Currently, the Universe of known FCCs comprises 14,153 FCCs that are included in at least one of the two databases (Figure 1).

Human biomonitoring: The measurement of the body burden of chemicals or their metabolites. Samples that are typically analyzed include urine, blood, plasma, and serum, but also saliva, breast milk, hair, nails, and other human tissues.

Exposome: This term comprises the totality of chemical, biological, and physical exposures that individuals encounter over their lifetimes. While the measurement of chemical exposures in biological samples can in theory be rather straightforward, physical exposures such as heat or noise are more difficult to quantify directly. In the context of this protocol, we only refer to chemical exposures.

Metabolome: The term describes all small molecules that are detected in a biological sample. These small molecules are either endogenously produced by the organism (e.g., amino acids, sugars, fatty acids) or taken up from exogenous sources (e.g., pesticides, drugs, FCCs).

Monitored vs. detected: Samples are often analyzed by a targeted approach to determine whether or not a specific chemical is present. In these cases, the chemical is monitored in the sample, but not necessarily detected. Only if the analytical method clearly confirms the presence of the chemical, it is considered detected.

Metabolites and parent compounds: Metabolites are the products of biochemical reactions in organisms. A metabolite is typically a small molecule derived from another molecular structure, the parent compound. Metabolites are targeted in biomonitoring studies if the parent compounds are known to be quickly converted in the organism. Depending on the source, the term biomarker may be used instead of metabolite. A specific metabolite provides evidence for exposure to one specific parent compound. In some cases, the same metabolite can be formed from structurally similar parent compounds. The detection of such metabolites typically does not allow the unambiguous identification of a specific parent compound, but rather a group of related chemicals. These metabolite are designated as unspecific. In other cases, metabolites are plausible but have not been experimentally proven. Such metabolites are designated as potential/predicted.

3.2 Authors' contributions and scientific advisory group

All planning steps, the scoping exercise and outcomes of pilot searches were discussed regularly within the core team (BG, LVP, KJG, CDK, MVM, OVM, LZ, JM). Authors contributed their specific expertise to the development of the protocol: food contact chemicals, materials, and articles (BG, KJG, MVM, LZ, JM), research synthesis methods (OVM), literature search and databases (JD, BG, KJG, LP, OVM). BG and LVM conducted pilot literature searches, refined the literature search strategy, and designed the data recording template. BG wrote the original draft of the protocol. All co-authors reviewed the draft and provided constructive improvements of the protocol.

The scientific advisory group gave regular input during this process and its members supported the core team with their specific expertise. The scientific advisory group will continue their support during the entire ongoing project. Members of the scientific advisory group are Jonathan Chevrier (McGill University, Canada), Barbara Demeneix and Jean Baptiste Fini (CNRS (French National Research Center), France), Jane Houlihan (Healthy Babies, Bright Futures, USA), Pete Myers (Environmental Health Sciences, USA),

Alex Odermatt (University of Basel, Switzerland), Katie Pelch (University of North Texas, USA), Rob Sargis (University of Illinois, USA), Verena Schreier (University of Basel, Switzerland), Emma Schymanski (University of Luxembourg, Luxembourg), Leo Trasande (New York University, USA), Laura Vandenberg (University of Massachusetts Amherst, USA), and Martin Wagner (Norwegian University of Science and Technology, Norway).

3.3 Research question

Initially, we assigned the key elements of the research question (Table 1) following the structure of a Population-Outcome (PO) question. PO questions are typically used when investigating a specific descriptive parameter for a population and are important for exposure assessments (Aiassa et al., 2015; James et al., 2016).

Table 1. Key elements of the research question.

**Question**	**Population (P)**	**Outcome (O)**
Which known FCCs have been monitored in the human body?	Human samples, such as blood, urine, hair, and breast milk, from people of any age, gender, or ethnicity	Any result describing the monitoring and/or detection of a known FCC or its metabolite

3.4 Scoping exercise

A scoping exercise was performed to get a sense of the human biomonitoring data that are available in different databases and the primary literature. Due to the high number of known FCCs, it was particularly important to decide *a priori* how these thousands of FCCs could be compared to chemicals that have been monitored in humans, whether a prioritization strategy was needed, which data should be extracted from the different sources, and how we could link information available in the database to the respective original sources.

During the scoping exercise, a tiered strategy was developed to obtain a comprehensive but not exhaustive overview of FCCs monitored in humans. Specifically, different approaches will be implemented as illustrated in Figure 2 and are further detailed in paragraphs 4 and 5. Based on this information, the evidence for FCCs in humans will be mapped and a database of **FCC**s **mon**itored in **hu**mans (**FCChumon**) will be compiled.

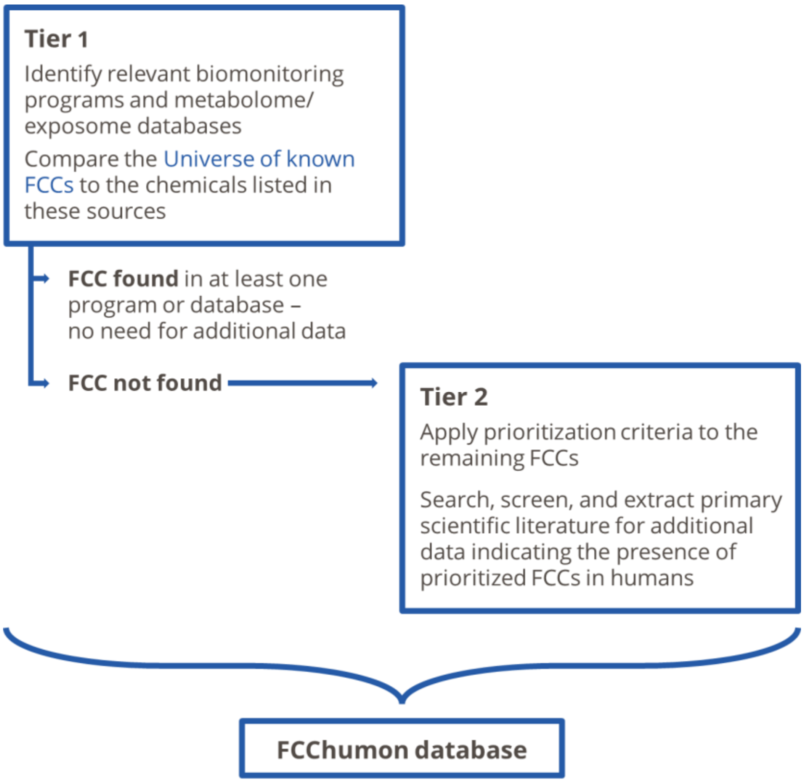

Figure 2. Tiered strategy to compare the Universe of known FCCs to biomonitoring programs and exposome/metabolome databases (tier 1) and to systematically map the evidence for the presence of FCCs in humans in the scientific literature (tier 2). Together, the results will be the basis of the Database of food contact chemicals monitored in humans (FCChumon).

3.5 Consultation process

Regular consultations within the core team and with the scientific advisory group led to the development of the tiered strategy. In particular, the following considerations were discussed and evaluated during the scoping exercise:

The high number of FCCs that form the Universe of known FCCs made it necessary to not only refer to the primary literature as an information source, but also consider established databases, i.e., tertiary sources (Virginia Tech University Libraries), that provide curated scientific information about the presence of chemicals in humans, as well as data from biomonitoring programs. The decision to integrate primary and tertiary sources will help to streamline the workflow and avoid duplication of effort. We are aware that this strategy does only partially follow the recommendations for scoping reviews and systematic evidence maps (Tricco et al., 2018). Therefore, we will refer to the term “evidence mapping” when describing the overall strategy and use the term “systematic evidence mapping” only for tier 2.

In order to combine the evidence from the different sources in an efficient way, we developed a tiered strategy. Tier 1 will be based on existing compilations of data that already contain comprehensive and detailed information about chemicals monitored in human samples. Linking these tertiary sources to the Universe of known FCCs will

enable a quick identification of relevant FCCs. We will consider the identification of an FCC in any of these sources as sufficient level of evidence for their presence in humans.

After we provisionally piloted the workflow as described 5.1, these pilot runs have shown that approximately 70% of the FCCs are listed in at least one of the sources applied in tier 1 (Figure 3). However, more than 4000 FCCs were not found in tier 1 (Table 2), which either means that the FCCs were targeted in human samples but never detected, or they were never monitored at all. Pilot searches of the primary literature for selected FCCs supported our hypothesis that the information sources used in tier 1 miss several FCCs for which further scientific evidence exists. Therefore, we decided to include searches of the primary literature in a second tier and systematically map the evidence for individual FCCs that were not found in tier 1 (Figure 2). For each chemical, multiple literature searches and screening of the search results will be needed, which can quickly multiply to several thousand individual literature data sets that become unmanageable. This called for a prioritization process that will allow us to focus on FCCs that are likely to result in human exposure based on the information of the FCCdb and the FCCmigex. The protocol for tier 2 will be applied to the prioritized group of chemicals in the first instance.

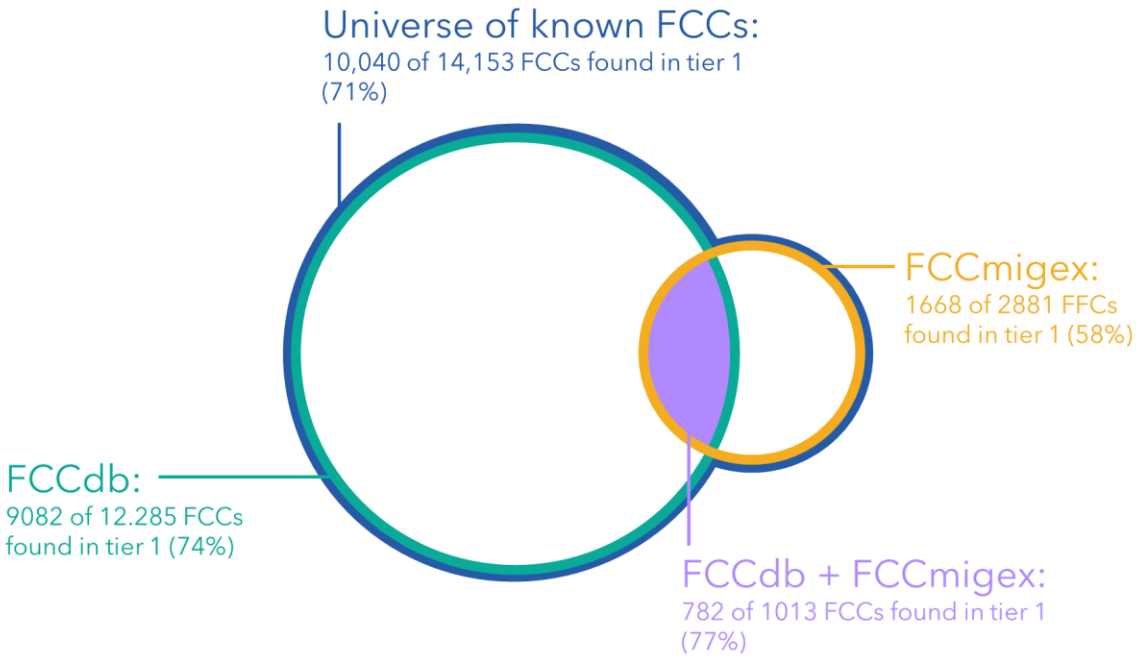

Figure 3. Schematic overview of FCCs with evidence for having been monitored in humans based on preliminary results of tier 1. Underlying data retrieved from Groh et al. (2021) and Geueke et al. (2022).

Prioritization as defined in this context is a decision tool that typically results in different outcomes and strongly depends on the criteria identified *a priori*, their application, and expert judgement. During the scoping exercise, we applied different prioritization criteria (and combinations thereof) to the >4000 FCCs that were not found in tier 1 (Table 2). This exercise helped to get an estimate of the expected numbers of FCCs and to discuss the relevance of the applied criteria based on concrete lists of FCCs.

First, we compared the percentage of tier-1 FCCs in terms of their listing in the FCCmigex and/or FCCdb (Figure 3). The FCCmigex includes FCCs that have evidence of or the potential for chemical migration into foods. These FCCs are likely to result in human exposure, but it is remarkable that over 40% of the chemicals in the FCCmigex are not listed in any biomonitoring program or metabolome/exposome database, compared to the 26% not listed in the FCCdb. This observation may be an indication for data gaps, e.g., caused by the presence of many NIAS in the FCCmigex, which could be filled or confirmed in tier 2. Therefore, we decided to prioritize the FCCs that have been detected in migrates and/or extracts of FCMs/FCAs (i.e., are included in the FCCmigex).

Then, we applied further prioritization criteria to the 1140 FCCs to make this number more manageable (Table 2) and discussed the strengths and weaknesses of each approach:

- A higher number of database entries for a given FCC in the FCCmigex is associated with a higher level of evidence of migration into food or a higher potential for such migration. Table 2 shows different options depending on the number of database entries per FCC (**1a-e**), which will allow the selection of a manageable list of prioritized FCCs.
- Additional filtering for FCCs that have been analyzed and detected in migrates, and not only in extracts increases the focus on FCCs with higher evidence for human exposure (**2**). However, by excluding FCCs detected only in extracts, we would miss potential links between FCCs that can theoretically migrate and their presence in humans.
- FCCs that are listed in the FCCmigex and FCCdb (**3a-b**) have additional evidence for intentional use in FCMs/FCAs. However, if we would focus on the overlap of FCCmigex and FCCdb only, we would miss many NIAS that have been detected in migrates and/or extracts.

Based on these scenarios, we decided to prioritize FCCs that were detected in migrates and/or extracts of FCMs/FCAs, regardless of their presence in the FCCdb. Further, we will start with the FCCs that have at least five database entries in the FCCmigex (**1e**), which means that the prioritized chemicals cover those FCCs with a high level of evidence of migration into food or a higher potential for such migration. In the future, we may then extend the list of FCCs analyzed in tier 2 by lowering the number of required FCCmigex entries per FCC (**1a-d**).

Table 2. Application of different prioritization criteria to FCCs that were not identified in tier 1. The reported numbers are the results of a pilot run of tier 1 and thus preliminary.

Possible prioritization criteria		Preliminary number of FCCs
	FCCs not identified in tier 1	4113
	**AND listed in**	
**1a**	FCCmigex<br>(filter: detection “yes”, >1 database entry per FCC)	1140
**1b**	FCCmigex<br>(filter: detection “yes”, >2 database entries per FCC)	467
**1c**	FCCmigex<br>(filter: detection “yes”, >3 database entries per FCC)	249
**1d**	FCCmigex<br>(filter: detection “yes”, >4 database entries per FCC)	165
**1e**	FCCmigex<br>(filter: detection “yes”, >5 database entries per FCC)	112
**2**	FCCmigex<br>(filters: detection “yes”, type of experiment: “migration into food” + “migration into food simulant”; >1 database entry per FCC)	563
**3a**	FCCdb + FCCmigex<br>(filter: detection “yes”)	227
**3b**	FCCdb + FCCmigex<br>(filters: detection “yes”, type of experiment: “migration into food” + “migration into food simulant”)	150

4 Information sources

The following biomonitoring programs, databases, and literature sources were identified during the scoping exercise and will be included.

4.1 Biomonitoring programs

We selected five biomonitoring programs that encompass a broad range of different chemicals, including known FCCs, and have a wide geographic coverage.

- National Health and Nutrition Examination Survey (NHANES) (Centers for Disease Control and Prevention, 2021)
- Canadian Health Measures Survey (CMHS) (Health Canada, 2021)
- European Human Biomonitoring Initiative (HBM4EU) (HBM4EU, 2022; IPCHEM, 2022)
- Korean National Environmental Health Survey (KoNEHS) (Jung et al., 2022)
- Biomonitoring California (Biomonitoring California, 2022)

Other major human biomonitoring programs in chemical exposure assessment were also reviewed but not included because they frequently monitored chemicals specific to non-FCM sources (e.g., pesticides) or the same chemicals that are targeted in the selected biomonitoring programs (Choi et al., 2015; World Health Organization, 2015).

4.2 Metabolome/exposome databases

Additionally, three databases were chosen that contain comprehensive information about chemicals that have been monitored in humans. These databases either refer to the metabolome or exposome of humans and/or mammals. They were selected after consulting with scientific experts and information specialists, and after supplementary in-house research.

- Human Metabolome Database (Wishart et al., 2022)
  The Human Metabolome Database (HMDB) is freely available and compiles information about small molecules found in the human body. It contains chemical, clinical and molecular biology/biochemistry data for over 250,000 chemicals.
- Blood Exposome Database (Barupal & Fiehn, 2019)
  The Blood Exposome Database (BlExpDB) is a collection of chemical compounds and related information associated to chemicals in the blood of mammals. The information was automatically extracted by text mining the content of PubMed and PubChem databases.
- Exposome Explorer (Neveu et al., 2019)
  Exposome-Explorer (Exp-Exp) is the first database dedicated to biomarkers of exposure to environmental risk factors for diseases. It aims to provide comprehensive data on all known biomarkers of exposure to dietary factors, pollutants, and contaminants monitored in population studies.

4.3 Bibliographic databases

During the scoping exercise, a significant part of all known FCCs was identified in biomonitoring programs and/or metabolome/exposome databases. However, other FCCs that have been frequently detected in migrates and extracts of FCMs were not found. Therefore, we developed a strategy that allows us to search and screen the primary literature for FCCs that were not identified in the above-mentioned sources. The following databases will be used to search for specific FCCs monitored in humans:

- PubMed
- Web of Science Core Collection (WoS)
- ScienceDirect
- CAS SciFinder^n

5 Methods

The tiered approach illustrated in Figure 2 applies different methods to map the available evidence on FCCs monitored in humans. Table 3 provides an overview of the steps that will be applied to investigate the three different types of information sources and how these are assigned to the two tiers.

Table 3. Overview of the proposed workflow to map the evidence for FCCs monitored in humans.

Tier 1		Tier 2
**Biomonitoring programs**	**Metabolome and exposome databases**	**Primary literature**
• Export/copy names and CAS IDs of chemicals that have been monitored in the five biomonitoring programs<br>• *If applicable:* Identify pairs of parent compounds and metabolites<br>• *If applicable:* Include whether the chemical has been detected in at least one of the biomonitoring samples, has never been detected or whether this is unknown	• Export chemical names, CAS IDs, and, if needed, further information and identifiers, from three metabolome/ exposome databases	• Define eligibility criteria<br>• Identify FCCs that were not found in tier 1 and apply prioritization criteria to these FCCs<br>• Run literature searches<br>• Manage and screen the literature<br>• Extract data and apply data coding strategy
Compare these lists to the Universe of FCCs		
Integration of results		

5.1 Tier 1: Comparing the Universe of known FCCs with tertiary sources

The Universe of known FCCs will be compared with chemicals included in national biomonitoring programs and/or listed in metabolome and exposome databases. Chemical information will be exported or copied from the sources and databases mentioned in 4.1 and 4.2 and the data will be stored, edited, formatted, and handled using Microsoft Excel or Microsoft Access.

5.1.1 Biomonitoring programs

Chemicals that have been included in the biomonitoring programs over the indicated time periods will be exported into a Microsoft Excel file. In some cases, chemicals are quickly converted into specific metabolites in the human body. These metabolites typically serve as indicators for human exposure to the parent compounds and are targeted in biomonitoring studies. In such cases, the analyzed metabolites will be paired with the parent compounds based on the information provided in the biomonitoring programs.

If available, Chemical Abstract Service identifiers (CAS IDs) will be exported from the original sources or, alternatively, the CAS IDs will be assigned to the exported chemicals.

Detection frequencies may be recorded. The CAS IDs of all FCCs that are part of the Universe of known FCCs will be compared to the chemicals listed in the five different biomonitoring programs (including parent compounds and metabolites).

5.1.2 Metabolome and exposome databases

For the HMDB, BlExpDB, and the Exp-Exp, we will download the most current version of each database and use these files for comparisons with the Universe of known FCCs. Since each database is different regarding content and structure, we will apply the following steps before running the comparisons:

- The HMDB contains detailed information about around 250,000 small molecule metabolites found in the human body. The database provides four filter options: “metabolite status”, “biospecimen”, “origin”, and “cellular location”. We will filter the HMDB by “metabolite status” only and will select the options “detected and quantified”, “detected but not quantified”, and “expected but not quantified”. The option “predicted” will not be included because pilot comparisons showed no overlaps of predicted metabolites to the Universe of known FCCs. By applying this filter, the number of metabolites was reduced to approximately 120,000.
- We will export all 65,957 chemicals that are listed in the BlExpDB. These chemicals were identified based on unique InChIKeys. Additionally, the database contains Canonical SMILES as chemical identifiers, but no CAS IDs. Therefore, we will extend the Universe of known FCCs by InChIKey and Canonical SMILES, if available, and use these identifiers for comparison to the BlExpDB.
- All 1212 chemicals listed in the Exp-Exp will be exported and compared with the Universe of known FCCs based on the available CAS IDs.

During the scoping exercise, we ran individual searches for known pairs of FCC parent compounds and metabolites. In all cases, the parent compound was listed in the tested databases. Therefore, we did not integrate further information on (possible) FCC metabolites before comparing these sources to the Universe of known FCCs.

5.2 Tier 2: Mapping further evidence for targeted FCCs

Based on systematic searches of the primary literature, tier 2 will provide additional evidence for individual FCCs, monitored in humans. This strategy is generally applicable to all FCCs, but it will only be applied to a selected set of prioritized chemicals in the first instance (see 3.5).

5.2.1 Prioritization of FCCs

All FCCs in the Universe of known FCCs that will not be identified in tier 1 will be selected for further prioritization to keep this stage manageable (Figure 2). The following criteria will be applied as a pragmatic solution to prioritize FCCs with a certain level of evidence for migration and/or extraction:

- The FCC is not identified in tier 1 and has at least 5 database entries representing its detection in different migration and extraction experiments as reported in the FCCmigex database (Table 2, **1e**).

For all prioritized FCCs, the possibility of missing any evidence for their presence in humans will be decreased by individual literature searches. In the future, the set of prioritized chemicals can be extended by applying other criteria, such as the hazard properties, the production volume, or the use in different FCMs. It is also possible to further combine prioritization criteria to select more specific groups of FCCs (e.g., by filtering for FCCs that were detected in migration experiments).

5.2.2 Eligibility criteria

Individual literature searches will be run for each prioritized FCC. The criteria defining whether a scientific study is eligible to be included or needs to be excluded are shown in Table 4. Studies will only be included if the sample originates from a human specimen and if the FCC for which the reference was found was monitored in these samples. The literature will be screened in a two-step process: first by titles and abstracts, then by full texts.

During title and abstract screening, it is typically not possible to find out all chemicals that were monitored in a study. Therefore, we will include all studies that report the measurement of chemicals in human specimens, without the requirement to identify individual FCCs in the titles and abstracts. In contrast, during full text screening, the eligibility criteria will only be applied with respect to the FCC(s), and/or to clearly related metabolite(s), for which the literature search was run, i.e., other chemicals that may have been monitored are not relevant in this context. If an FCC was monitored but not detected the study will also be included.

No date and language restrictions will be applied. Inclusion will be limited to primary research articles showing original research data. Review articles, conference abstracts, presentations, dissertations, books and book chapters will be excluded.

Table 4. Eligibility criteria for the title and abstract and full-text screening steps.

Screening step		Inclusion criteria	Exclusion criteria
**Title and Abstract**	**P**	• Analyzed sample originates from a human specimen (e.g., blood, urine, breast milk).<br>• Volatile organic compounds analyzed (e.g., in breath or on human skin).<br>• No restrictions on the sample in terms of the collected tissues or fluids/gases excreted.<br>• No restrictions on age, sex, or life stage.	• Analyzed sample originates from species other than human, is an environmental sample, or undefined.<br>• Analyzed sample is derived from human cell culture.
	**O**	• Chemical is monitored (in the human specimen).	• The biomonitoring study concerns measurements that are related to chemical exposure (e.g., exposure-related effects), but does not use chemical analysis methods to confirm the presence of the chemical.<br>• Only internal biomolecules are measured (e.g., glucose, cholesterol, proteins).<br>• Only pharmaceuticals, drugs, and dietary supplements are measured.<br>• Chemicals are measured in humans after accidental poisoning or intentional administration.
**Full text**	**P**	• Analyzed sample originates from a human specimen (e.g., blood, urine, breast milk).<br>• No restrictions on the sample in terms of the collected tissues or fluids/gases excreted.<br>• No restrictions on age, sex, or life stage.	• Analyzed sample originates from species other than human, is an environmental sample, or undefined.<br>• Analyzed sample is derived from human cell culture.
	**O**	• At least one FCC for which the reference was found in the literature search or clearly related metabolite(s) are monitored (in a human specimen).<br>• FCC or its metabolite(s) is/are identified with at least Level 2 confidence (“probable structure”).*	• The FCC is mentioned in the full-text in another context (e.g., as solvent, internal standard).<br>• The biomonitoring study concerns measurements that are related to FCC exposure (e.g., exposure-related effects), but does not use chemical analysis methods to confirm the presence of the chemical.<br>• FCC is measured in humans after accidental poisoning or intentional administrations.

*Based on the framework for communicating confidence when identifying small molecules (Schymanski et al., 2014). P = Population, O = Outcome.

5.2.3 Search strategies

A single FCC can have dozens of different synonyms, including highly systematic but rarely used names as well as generic and trade names. In the FCCdb and FCCmigex, chemical names were assigned based on their common uses in the underlying source references. We will query PubMed, WoS, ScienceDirect, and SciFinder^{n} for these chemical names and/or the respective CAS IDs of the FCCs (Table 5). To further specify the searches, we will always combine the chemical identifier with keywords related to human biomonitoring. The search strings and settings will be slightly adapted to fit the requirements of the respective databases.

In pilot searches, we applied these search strings to twelve of the 112 preliminary prioritized FCCs (see 3.5 and 5.2.1). Overall, the resulting number of hits matched our expectations regarding content and quantity in all four databases. Only the searches for CAS IDs in PubMed resulted in fewer and less relevant results when compared to the searches for chemical names. Therefore, we will not include searches for CAS IDs in PubMed.

For four of the twelve selected chemicals, we were aware of scientific papers reporting their measurements in humans before starting the literature search. With the exception of one, we found these papers in at least one of the databases by applying the search strategies shown in Table 5. The paper that was missed used another synonym for the chemical "PET cyclic trimer" and the CAS ID of this chemical was not found in CAS SciFinder^{n} although the paper is listed (Diamantidou et al., 2022). In case of no hits for a specific FCC, such limitations may be overcome by omitting the quotation marks used around the chemical name. We will not include this option systematically but will consider it if the first searches did not result in any results.

Pilot searches asking for the CAS ID instead of the chemical name and selecting the search field *EC/RN number* in PubMed resulted in fewer and less relevant results than a search asking for the chemical name in *all fields* instead. Therefore, we decided to search PubMed for the chemical name only.

In CAS SciFinder^{n}, pilot searches showed that integrating the term "human" resulted in too many unspecific hits. Therefore, and in contrast to the searches in the other databases, we did not include this search term.

Table 5. Search strategies for PubMed, WoS, ScienceDirect, and CAS SciFinder^{n}

Database	Chemical identifier	Operator	Search string:<br><br>Key words related to human biomonitoring	Further settings/filters
PubMed	"chemical name"	AND	(human OR blood OR urine OR serum OR hair OR nail OR plasma OR biomon* OR "breast milk")	• Search *all fields*<br>• No filters applied
WoS	"chemical name"	AND	(human OR blood OR urine OR serum OR hair OR nail OR plasma OR biomon* OR "breast milk")	• Advanced search<br>• Search *all fields*<br>• Document type: Article
ScienceDirect	"chemical name"	AND	(human OR blood OR urine OR serum OR hair OR nail OR plasma OR biomonitoring OR "breast milk")	• Advanced search<br>• Search “chemical name” in *full text*<br>• Search all other keywords in *title, abstract or author-specific keywords*<br>• Article type: Research article
CAS SciFinder^{n}	CAS ID	AND	(blood OR urine OR serum OR hair OR nail OR plasma OR biomon* OR "breast milk")	• Searching for References<br>• Search keywords in the main search field<br>• Search CAS ID in *CAS Registry Number*<br>• Filter for document type: Journal

5.2.4 Management and screening of the literature

For each FCC, the search results from four scientific databases will be transferred into a separate Endnote library and duplicates will be removed (Figure 4). We will then upload all individual Endnote libraries to the freely available online tool *Cadima* (Kohl et al., 2018). There, identifiers will be automatically assigned to each reference and kept throughout the project.

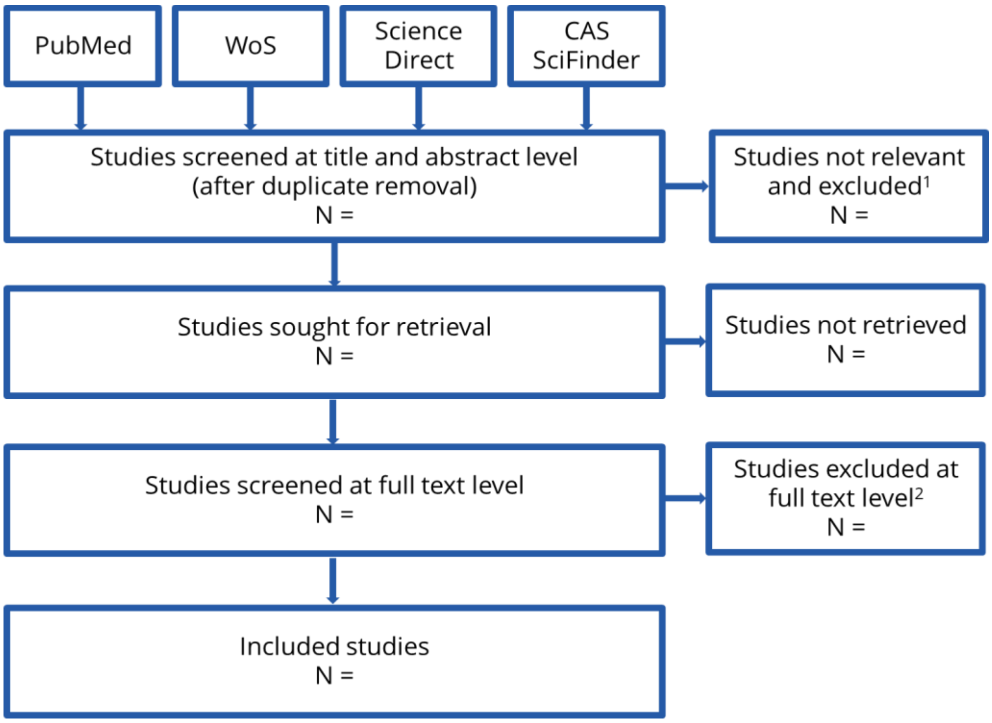

Figure 4. Example workflow of tier 2. Individual literature searches will be run for each prioritized FCC. [1]Clearly irrelevant studies will be excluded during title and abstract screening. [2]Reasons for exclusion may be: full text not accessible, no primary data presented, eligibility criteria not fulfilled.

If several FCCs were monitored in one study, this reference may appear in different FCC-specific Endnote libraries and eventually lead to duplicates in *Cadima*. To remove these duplicates but keep the information about the FCCs for which the reference was found, we will perform the following steps before starting the title and abstract screening:

- The merged reference list will be downloaded from *Cadima* (including ALL duplicates) and saved in an Excel file.
- Copies of the same references will be searched based on the DOI and the article title.
- References that appear more than once will be merged into one entry. For each of these merged entries, we will make sure to add information about the FCCs for which the reference was found (names and CAS IDs) as well as all alternative *Cadima* identifiers of further copies of the same reference.
- Based on this list, all duplicates will be removed from *Cadima* before title and abstract screening.

The retained references will be screened first at title and abstract level, then at full text level in *Cadima* by applying the eligibility criteria detailed in Table 4.

During both screenings phases, ten percent of entries will be screened by two reviewers in parallel, and discrepancies will be resolved bilaterally or by consulting the core team. If needed, the eligibility criteria will be refined before proceeding with the screening of the remaining studies. Clearly irrelevant studies will be excluded during title and abstract screening. All other studies that will be included during title and abstract screening and for which full texts are available will undergo full text screening by applying slightly modified and more specific eligibility criteria (Table 4). The reasons for exclusion during full text screening will be recorded.

During pilot runs of the full text screening, we realized that the decision on the Outcome (i.e., were any of the FCCs for which the reference was found monitored in the study?) required a rather detailed analysis of the reference. Therefore, we decided to combine the full text screening and the data extraction.

Figure 5 illustrates this combined workflow in detail:

1. Formal exclusion criteria (e.g., full text not accessible, full text not assessable, no primary data presented) will be evaluated and documented in *Cadima*.
2. We will check whether the inclusion criteria related to the Population will be fulfilled (Table 4). A NO to this question will also result in a NO for the Outcome because this is directly related to the investigated Population. In such a case the reference will be excluded.
3. If the inclusion criteria for the Population are fulfilled, we will investigate the eligibility criteria for the Outcome. If at least one FCC that is linked to the reference was monitored, the reference will be included.
4. For each reference, the decisions on inclusion and exclusion will be documented in *Cadima*.
5. For each FCC that was monitored in the reference, we will directly complete the data extraction process in *SciExtract* (see also Table 6).

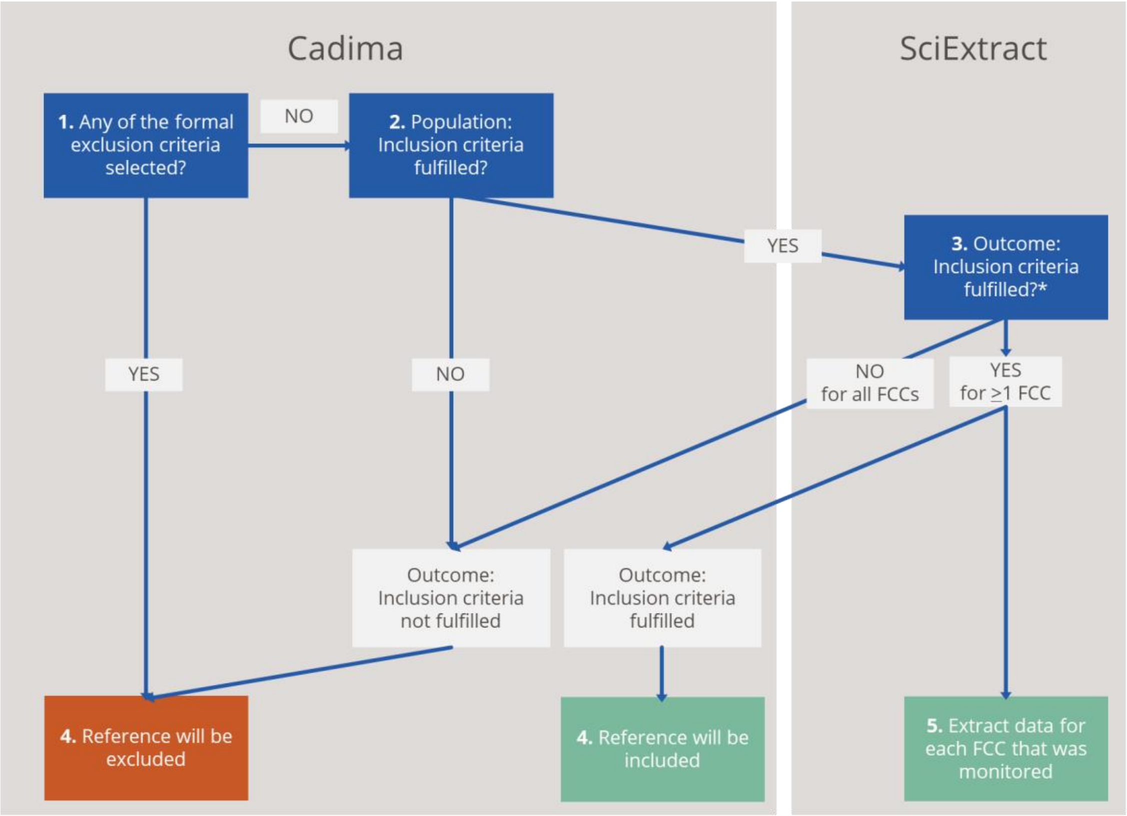

Figure 5. Scheme of the workflow combining full text screening in *Cadima* and data extraction in *SciExtract*. *The Outcome element of the research question is directly related to question 1 of the data extraction in *SciExtract*.

5.2.5 Data coding and extraction

Data will be extracted from the eligible full text references by using the tool *SciExtract*, which was developed to allow systematic, safe, and reproducible data extraction for the FCCmigex database (Geueke et al., 2022). For the FCChumon database, we will set up a new data extraction template in *SciExtract* as detailed below. Ten percent of references that fulfill the inclusion criteria of the full text screening will be extracted by two independent reviewers. Reviewer pairs will compare their results and resolve any conflicts. If needed, they can ask for a third opinion within the core team. During the whole data extraction process, it will be possible to discuss uncertainties relevant to data extraction by calling for a second opinion.

The bibliographic information and the FCCs that were related to this reference will already be provided as a result of the literature management process. During full-text screening, we will check which of the related FCCs were monitored in human samples (Outcome in *Cadima* and question Q1 in *SciExtract*) (Figure 5). For each of the monitored FCCs, we will answer different questions by selecting pre-coded options or entering free text as illustrated in Table 6. The collected data will provide information on the FCC, the type of analyte (Q2), the human specimen (Q4), and whether the FCC was detected in the sample or not (Q5) (Table 6). Furthermore, the type of analytical approach (Q6) and additional information on the analyte will be documented (Q3).

Table 6. Data coding and extraction, tier 2. For each monitored FCC, the answer to question 1 will be YES and question 2-6 need to be answered separately. Several database entries can therefore be generated for one reference.

Question	Data category	Data captured	Comments
	Bibliographic information	• Author(s)<br>• Year of publication<br>• Journal<br>• Title<br>• URL<br>• Volume<br>• Issue<br>• Pages<br>• DOI<br>• Abstract	
	FCC	• Chemical name (used in the literature search)<br>• CAS ID	The link between an FCC and a reference is the result of the individual literature searches.
Q1	Was the FCC monitored?	• Yes<br>• No	This information answers whether a reference will be included or excluded (Figure 5).<br>If "No", questions 2-5 will not be relevant.
Q2	Type of analyte(s)	• FCC<br>• Specific metabolite(s) of FCC<br>• Unspecific metabolite(s) of FCC<br>• Potential metabolite(s)	
Q2a	If analyte = FCC	• Synonym used in the reference	Optional<br>Free text allowed
Q2b	If analyte(s) = metabolite(s)	• Chemical name of the metabolite	Required, if metabolite<br>Free text allowed
Q2c	If analyte(s) = metabolite(s)	• CAS ID	Optional<br>Follow formatting of CAS IDs
Q3	Analyte is	• VOC<br>• Endogenous metabolite	Optional<br>Free text allowed
Q4	Human specimen	• Blood<br>• Urine<br>• Plasma<br>• Serum<br>• Hair<br>• Nail<br>• Breast milk<br>• Adipose tissue<br>• Amniotic fluid<br>• Cord blood	Controlled additions of terms allowed as needed

Question	Data category	Data captured	Comments
		• Unclear/unknown	
Q5	Detected in humans	• Yes<br>• No<br>• Unclear/unknown	
Q6	Type of analysis	• Targeted<br>• Non-targeted<br>• Unclear/unknown	

Depending on the type of analyte (Q2), further information may be needed: If the FCC was analyzed directly, synonyms may be entered in field Q2a. If the analyte is any kind of metabolite, it is required to enter the name of the metabolite (Q2b). Ideally the CAS ID of the metabolite will also be provided (Q2c).

5.2.6 Study quality assessment

The study quality will not be assessed.

5.3 Integration of results and reporting

The outcome of this project will be described in a manuscript that will undergo peer review. The key results will be described in a narrative form and visually supported by illustrations. Limitations that we may encounter in the course of tier 1 and tier 2 will be further detailed in the planned publication. Table 7 shows a view on how the data can be collected and stored. In addition, the data will be made available in an interactive dashboard so that users can search and filter for FCCs and related information. In a next step, the results will be linked to the FCCdb and FCCmigex databases. The FCCs monitored in humans, and those that were not found in any source, will be discussed in the context of the Universe of known FCCs. Example questions that may be answered based on the generated results are:

- Which and how many FCCs have been monitored in humans? Which and how many FCCs have been detected in humans?
- Which FCCs or groups of FCCs have been most frequently monitored in national biomonitoring programs, metabolome/exposome databases, or in the primary literature?
- Is there a correlation between the number of database entries for an FCC in the FCCmigex and the amount of evidence available from human biomonitoring studies?
- Which and how many of the tier-2 prioritized FCCs have been monitored in humans?
- Which FCCs detected in humans are listed in the FCCdb?
- How many and which FCCs monitored in humans are used in a specific FCM? How many and which FCCs detected in humans have been detected in migrates of a specific FCM?
- For which FCM types can we identify the biggest data gaps regarding biomonitoring data on chemicals used in/migrating from it?

Table 7. Possible overview of how to map the evidence for the presence of an FCC in human samples. The bullet points indicate answer options.

	Tier 1								Tier 2				
	**Biomonitoring programs**[1]					**Metabolome/exposome databases**[2]			**Evidence from tier 1?**[3]	**Prioritized?**[4]	**FCC or metabolite(s) monitored in humans?**[5]	**FCC or metabolite(s) detected in humans?**[6]	**Reference(s)**[6]
	**NHANES**	**CHMS**	**HBM4EU**	**KoNEHS**	**Biomon Cal**	**HMDB**	**BlExpDB**	**Exp-Exp**					
**FCC**	• FCC: Monitored and detected<br>• FCC: Monitored but never detected<br>• FCC: Monitored but only rarely detected or detection unclear<br>• Metabolite: Monitored and detected<br>• Metabolite: Monitored but never detected<br>• Metabolite: Monitored but detection unclear<br>• Not listed					• Detected and quantified<br>• Detected but not quantified<br>• Expected but not quantified<br>• Not listed	• Listed<br>• Not listed	• Listed<br>• Not listed	• Yes<br>• No	• Yes<br>• No	• Yes (specify specimen(s))<br>• No	• Yes<br>• No<br>• Not applicable	• Link to Reference(s)<br>• Not applicable

[1] For each of the five biomonitoring programs, one of the seven possible outcomes needs to be selected.
[2] If technically feasible, links will be set into the cells that direct to the FCC-specific entry in the respective database.
[3] "Evidence from tier 1?" = "Yes", if at least one column from tier 1 has an entry other than "Not listed". In that case the other columns of tier 2 remain empty.
[4] Only applicable if "Evidence from tier 1?" = "Yes"
[5] "Monitored in humans?" = "Yes" for all studies that were included after full text screening.
[6] Only applicable if "Monitored in humans?" = "Yes". Optional: include information on human specimen.

6 Financial Support

This protocol is part of the Food Contact Chemicals and Human health (FCCH) project. The FCCH project is funded by project-related grants from Sympany Foundation, MAVA Foundation, and Minerva Stiftung as well as by the Food Packaging Forum's (FPF) own resources from donations. All FPF funding sources are listed under https://www.foodpackagingforum.org/about-us/funding.

7 Declaration of Competing Financial Interests

The authors declare that no competing financial interests exist.

8 References

Aiassa, E., Higgins, J. P., Frampton, G. K., Greiner, M., Afonso, A., Amzal, B., Deeks, J., Dorne, J. L., Glanville, J., Lövei, G. L., Nienstedt, K., O'Connor A, M., Pullin, A. S., Rajić, A., & Verloo, D. (2015). Applicability and feasibility of systematic review for performing evidence-based risk assessment in food and feed safety. *Crit Rev Food Sci Nutr, 55*(7), 1026-1034. https://doi.org/10.1080/10408398.2013.769933.

Barupal, D. K., & Fiehn, O. (2019). Generating the Blood Exposome Database using a comprehensive text mining and database fusion approach. *Environ Health Perspect, 127*(9), 97008. https://doi.org/10.1289/ehp4713.

Biomonitoring California. (2022). Chemicals Biomonitored in California. Retrieved from: https://biomonitoring.ca.gov/chemicals/chemicals-biomonitored-california.

Centers for Disease Control and Prevention. (2021). Fourth national report on human exposure to environmental chemicals. Updated tables, March 2021: volume three https://stacks.cdc.gov/view/cdc/105344.

Choi, J., Mørck, T. A., Joas, A., & Knudsen, L. E. (2015). Major national human biomonitoring programs in chemical exposure assessment. *AIMS Environmental Science, 2*(3), 782-802. https://doi.org/10.3934/environsci.2015.3.782.

Diamantidou, D., Mastrogianni, O., Tsochatzis, E., Theodoridis, G., Raikos, N., Gika, H., & Kalogiannis, S. (2022). Liquid chromatography-mass spectrometry method for the determination of polyethylene terephthalate and polybutylene terephthalate cyclic oligomers in blood samples. *Analytical and Bioanalytical Chemistry, 414*(4), 1503-1512. https://doi.org/10.1007/s00216-021-03741-6.

Geueke, B. (2018). Dossier - Non-intentionally added substances. *Food Packaging Forum*. https://doi.org/10.5281/zenodo.126533.

Geueke, B., Groh, K. J., Maffini, M. V., Martin, O. V., Boucher, J. M., Chiang, Y.-T., Gwosdz, F., Jieh, P., Kassotis, C. D., Łańska, P., Myers, J. P., Odermatt, A., Parkinson, L. V., Schreier, V. N., Srebny, V., Zimmermann, L., Scheringer, M., & Muncke, J. (2022). Systematic evidence on migrating and extractable food contact chemicals: Most chemicals detected in food contact materials are not listed for use. *Critical Reviews in Food Science and Nutrition*, 1-11. https://doi.org/10.1080/10408398.2022.2067828.

Groh, K. J., Geueke, B., Martin, O., Maffini, M., & Muncke, J. (2021). Overview of intentionally used food contact chemicals and their hazards. *Environment International, 150*, 106225. https://doi.org/https://doi.org/10.1016/j.envint.2020.106225.

HBM4EU. (2022). EU HBM Dashboard. Retrieved from: https://www.hbm4eu.eu/what-we-do/european-hbm-platform/eu-hbm-dashboard/

Health Canada. (2021). Sixth report on human biomonitoring of environmental chemicals in Canada. Minister of Health, Ottawa, ON. https://www.canada.ca/content/dam/hc-sc/documents/services/environmental-workplace-health/reports-publications/environmental-contaminants/sixth-report-human-biomonitoring/pub1-eng.pdf.

IPCHEM. (2022). Human Biomonitoring Data Module: HBM4EU-aggregated - HBM4EU aggregated workbook by VITO. Retrieved from: https://ipchem.jrc.ec.europa.eu/#showmetadata/HBM4EUAGGREGATED.

James, K. L., Randall, N. P., & Haddaway, N. R. (2016). A methodology for systematic mapping in environmental sciences. *Environmental Evidence, 5*(1), 7. https://doi.org/10.1186/s13750-016-0059-6.

Jung, S. K., Choi, W., Kim, S. Y., Hong, S., Jeon, H. L., Joo, Y., Lee, C., Choi, K., Kim, S., Lee, K.-J., & Yoo, J. (2022). Profile of environmental chemicals in the Korean population - Results of the Korean National Environmental Health Survey (KoNEHS) Cycle 3, 2015-2017. *International Journal of Environmental Research and Public Health, 19*(2), 626. https://doi.org/doi:10.3390/ijerph19020626.

Kohl, C., McIntosh, E. J., Unger, S., Haddaway, N. R., Kecke, S., Schiemann, J., & Wilhelm, R. (2018). Online tools supporting the conduct and reporting of systematic reviews and systematic maps: a case study on CADIMA and review of existing tools. *Environmental Evidence, 7*(1), 8. https://doi.org/10.1186/s13750-018-0115-5.

Muncke, J., Andersson, A.-M., Backhaus, T., Boucher, J. M., Carney Almroth, B., Castillo Castillo, A., Chevrier, J., Demeneix, B. A., Emmanuel, J. A., Fini, J.-B., Gee, D., Geueke, B., Groh, K., Heindel, J. J., Houlihan, J., Kassotis, C. D., Kwiatkowski, C. F., Lefferts, L. Y., Maffini, M. V., Martin, O. V., Myers, J. P., Nadal, A., Nerin, C., Pelch, K. E., Fernández, S. R., Sargis, R. M., Soto, A. M., Trasande, L., Vandenberg, L. N., Wagner, M., Wu, C., Zoeller, R. T., & Scheringer, M. (2020). Impacts of food contact chemicals on human health: a consensus statement. *Environmental Health, 19*(1), 25. https://doi.org/10.1186/s12940-020-0572-5.

Nerín, C., Bourdoux, S., Faust, B., Gude, T., Lesueur, C., Simat, T., Stoermer, A., Van Hoek, E., & Oldring, P. (2022). Guidance in selecting analytical techniques for identification and quantification of non-intentionally added substances (NIAS) in food contact materials (FCMS). *Food Additives & Contaminants: Part A, 39*(3), 620-643. https://doi.org/10.1080/19440049.2021.2012599.

Neveu, V., Nicolas, G., Salek, R. M., Wishart, D. S., & Scalbert, A. (2019). Exposome-Explorer 2.0: an update incorporating candidate dietary biomarkers and dietary associations with cancer risk. *Nucleic Acids Research, 48*(D1), D908-D912. https://doi.org/10.1093/nar/gkz1009.

Schymanski, E.L., Jeon, J., Gulde, R., Fenner, K., Ruff, M., Singer, H.P., & Hollender, J. (2014) Identifying small molecules via high resolution mass spectrometry: communicating confidence. *Environmental Science & Technology*, *48*(4):2097-2098. https://pubs.acs.org/doi/10.1021/es5002105.

Tricco, A. C., Lillie, E., Zarin, W., O'Brien, K. K., Colquhoun, H., Levac, D., Moher, D., Peters, M. D. J., Horsley, T., Weeks, L., Hempel, S., Akl, E. A., Chang, C., McGowan, J., Stewart, L., Hartling, L., Aldcroft, A., Wilson, M. G., Garritty, C., Lewin, S., Godfrey, C. M., Macdonald, M. T., Langlois, E. V., Soares-Weiser, K., Moriarty, J., Clifford, T., Tunçalp, Ö., & Straus, S. E. (2018). PRISMA Extension for Scoping Reviews (PRISMA-ScR): Checklist and Explanation. *Ann Intern Med, 169*(7), 467-473. https://doi.org/10.7326/m18-0850.

Virginia Tech University Libraries. Primary, secondary, and tertiary sources - a guide from university libraries. Retrieved from: https://odyssey.lib.vt.edu/files/original/657260b510765575bbcb7674d7e783a1862c3244.pdf.

Warner, G. R., & Flaws, J. A. (2018). Bisphenol A and phthalates: How environmental chemicals are reshaping toxicology. *Toxicological Sciences, 166*(2), 246-249. https://doi.org/10.1093/toxsci/kfy232.

Wishart, D. S., Guo, A., Oler, E., Wang, F., Anjum, A., Peters, H., Dizon, R., Sayeeda, Z., Tian, S., Lee, B. L., Berjanskii, M., Mah, R., Yamamoto, M., Jovel, J., Torres-Calzada, C., Hiebert-Giesbrecht, M., Lui, V. W., Varshavi, D., Varshavi, D., Allen, D., Arndt, D., Khetarpal, N., Sivakumaran, A., Harford, K., Sanford, S., Yee, K., Cao, X., Budinski, Z., Liigand, J., Zhang, L., Zheng, J., Mandal, R., Karu, N., Dambrova, M., Schiöth, H. B., Greiner, R., & Gautam, V. (2022). HMDB 5.0: the Human Metabolome Database for 2022. *Nucleic Acids Res, 50*(D1), D622-d631. https://doi.org/10.1093/nar/gkab1062.

World Health Organization. (2015). Human biomonitoring: facts and figures. Copenhagen: World Health Organization. Regional Office for Europe. https://apps.who.int/iris/handle/10665/164588.
