# Supplementary material for: Evidence for widespread human exposure to food contact chemicals
Source: J Expo Sci Environ Epidemiol. 2024 Sep 17;35(3):330–41. doi: 10.1038/s41370-024-00718-2 (PMC12069106; doi:10.1038/s41370-024-00718-2)
Supplement: Supplementary file 2 — Supplementary information [file 41370_2024_718_MOESM2_ESM.pdf]

Supplementary information

Evidence for widespread human exposure to food contact chemicals

Birgit Geueke[1•], Lindsey V. Parkinson[1], Ksenia J. Groh[2], Christopher D. Kassotis[3], Maricel V. Maffini[4], Olwenn V. Martin[5], Lisa Zimmermann[1], Martin Scheringer[6,7] and Jane Muncke[1]

[1] Food Packaging Forum Foundation, Zurich, Switzerland

[2] Department of Environmental Toxicology, Eawag, Swiss Federal Institute of Aquatic Science and Technology, Dübendorf, Switzerland

[3] Institute of Environmental Health Sciences and Department of Pharmacology, Wayne State University, Detroit, MI, USA

[4] Independent Consultant, Frederick, MD, USA

[5] Department of Arts & Science, University College London, UK

[6] RECETOX, Masaryk University, Brno, Czech Republic

[7] Department of Environmental Systems Science, ETH Zurich, Switzerland

• corresponding author, email: birgit.geueke@fp-forum.org

Systematic evidence map (step 2)

Details of the data extraction process

Question	Answer options
Was the prioritized FCC monitored in a human sample?	Yes | No
Which type of human sample was analyzed?	Urine | Plasma | Serum | Blood | Hair | Nail | Breast milk | Adipose tissue| Amniotic fluid | Cord plasma | Cord serum | Cord blood | Breath | Saliva | Skin | Placenta | Other
Was the monitored FCC detected?	Yes | No | Unclear/unknown
What analytical method was applied?	Targeted | Non-targeted | Unclear/unknown
What type of chemical was analyzed?	FCC | specific metabolite of FCC | unspecific metabolite of FCC
If analyte = metabolite: What is the chemical name and CAS number of the metabolite?	(free text)

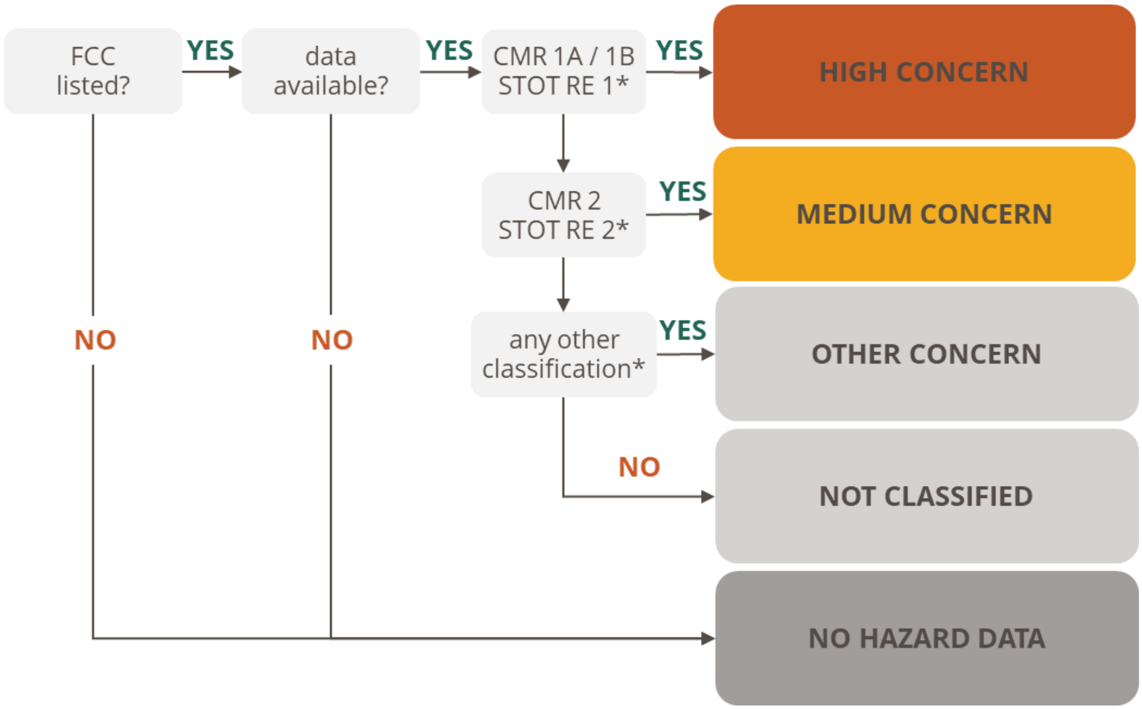

Figure S1. Flow scheme illustrating the process of assigning hazard data to FCCs as well as demonstrating the absence of such data. Hazard data were retrieved from the European Chemical Agency's (ECHA) Classification and Labelling Inventory aligned with the Globally Harmonized System (GHS) for chemical classification and labelling [40] and the GHS-aligned classifications by the Japanese Government [41]. *It was sufficient if this requirement was fulfilled for at least one hazard category - even in the absence of data for all other hazard categories.

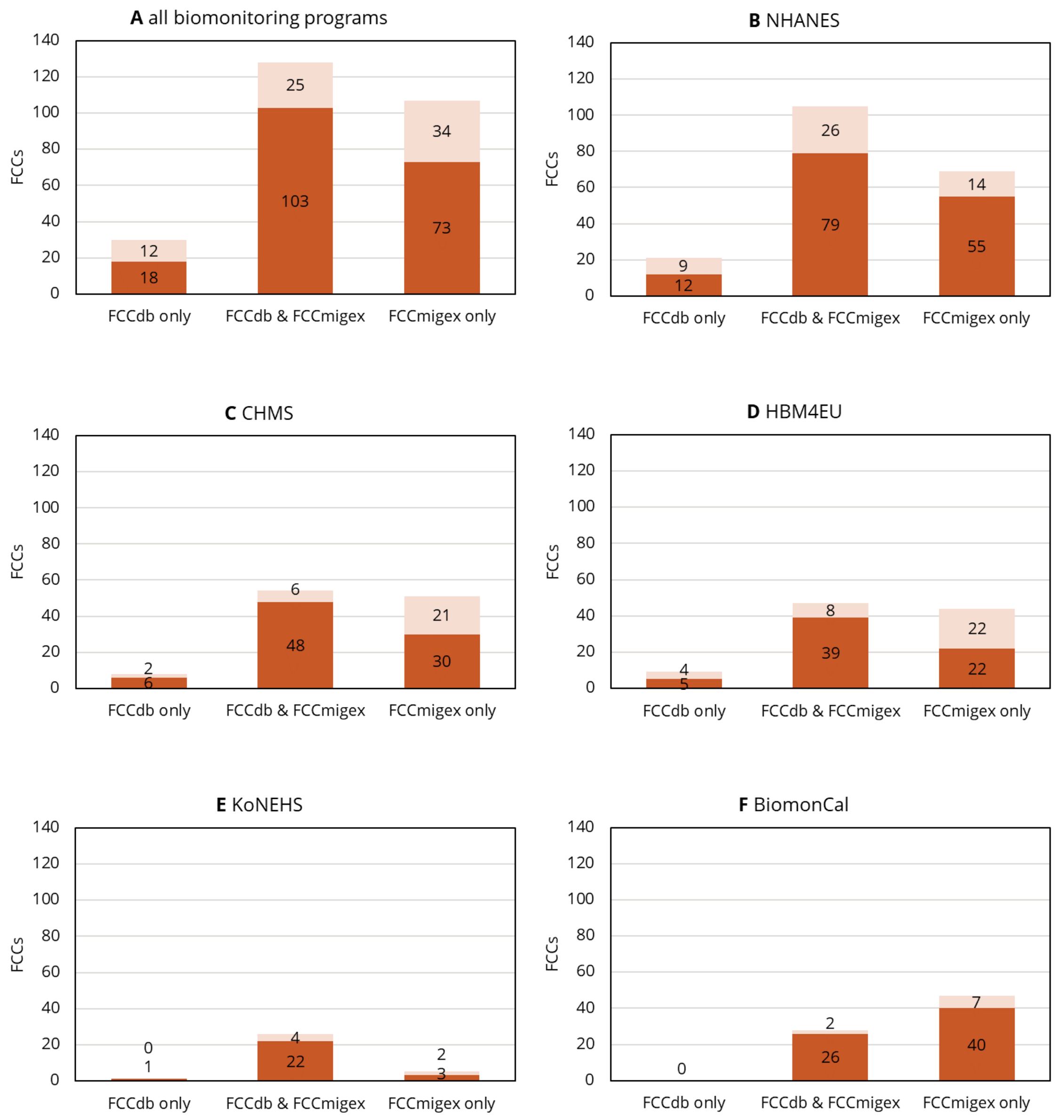

Figure S2. Number of FCCs in all biomonitoring programs (**A**), in NHANES (**B**), CHMS (**C**), HBM4EU (**D**), KoNEHS (**E**), and Biomonitoring California (**F**). The dark orange parts of the columns indicate the number of FCCs that have been detected in humans and the light orange parts represent the number of FCCs that have been monitored, but have not been detected. Each figure displays the number of FCCs that are included only in the FCCdb, only in the FCCmigex, and in both databases.

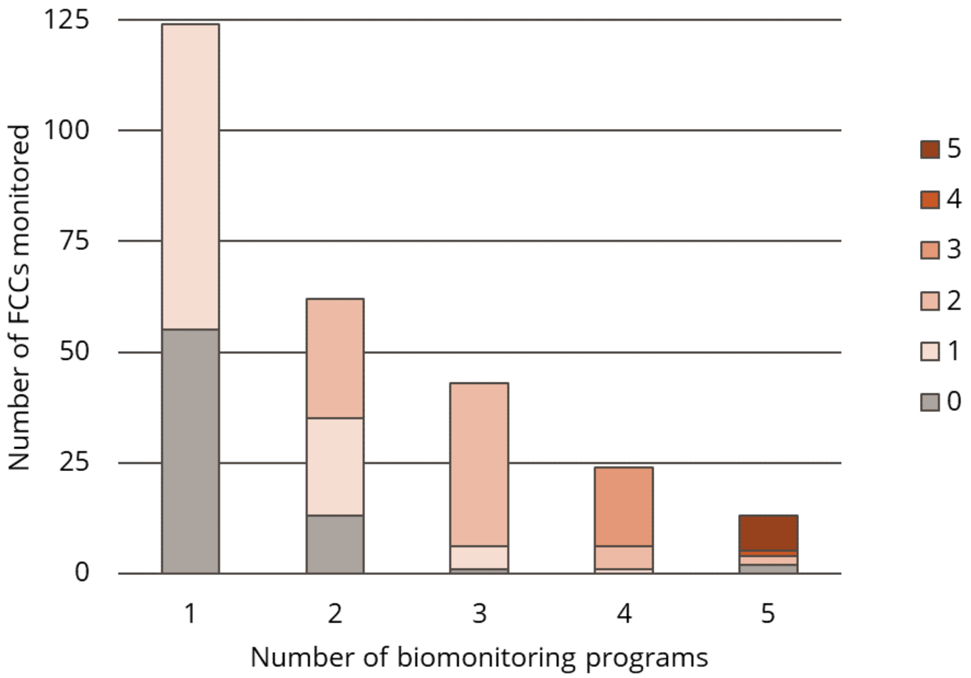

Figure S3. Number of FCCs monitored across multiple biomonitoring programs. The coloring scheme of the stacked columns illustrates how often chemicals have been monitored and detected across the different biomonitoring programs in which they have been included. The eight FCCs (or their metabolites) that have been monitored and detected across all five biomonitoring programs are arsenic (CAS 7440-38-2), benzyl butyl phthalate (CAS 8568-7), bisphenol A (CAS 80-05-7), cadmium (CAS 7440-43-9), di(2-ethylhexyl) phthalate (CAS 117-81-7), lead (CAS 7439-92-1), mercury (CAS 7439-97-6), and monobutyl phthalate (CAS 131-70-4).

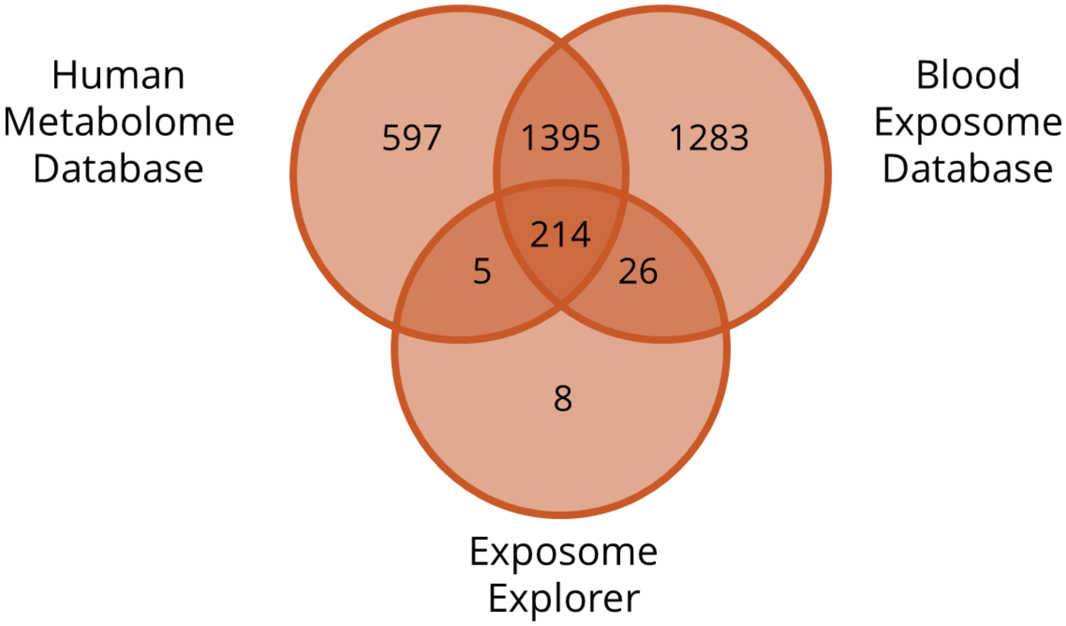

Figure S4. Number of food contact chemicals from the Universe of known FCCs that were present in the three metabolome/exposome databases and the respective overlaps.

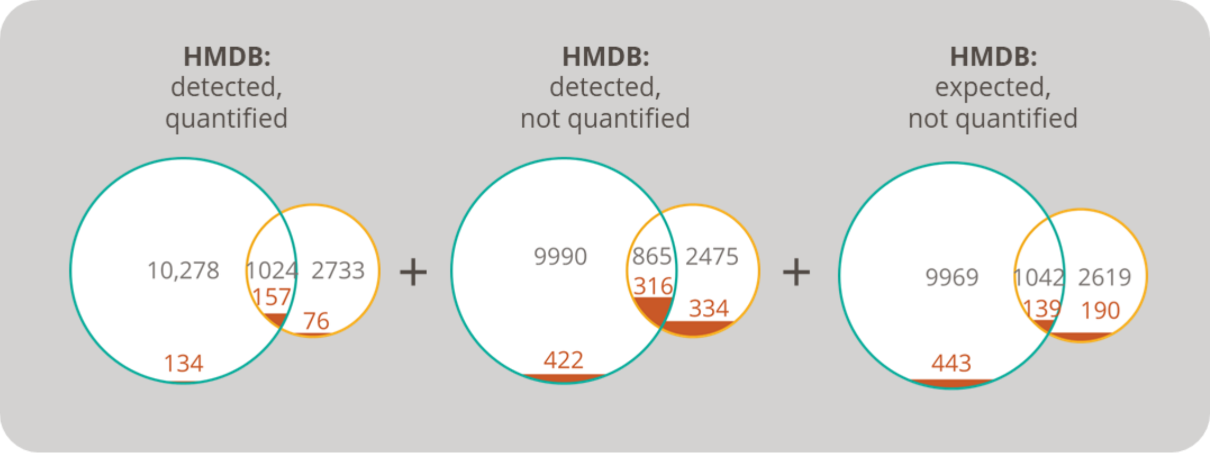

Figure S5. FCCs with evidence for presence in humans according to their "metabolite status" used in the HMDB. The green circle represents the FCCdb, the yellow circle the FCCmigex database. Evidence for presence of FCCs in humans is indicated by the orange filling of the respective areas. For any chemical labeled as "detected" in the HMDB, there is solid experimental evidence and data from the literature supporting its metabolite's existence and quantification. The evidence for "expected" metabolites is based on biochemistry, enzymology, or known constituents found in the human body, in combination with literature reviews and putative identification.

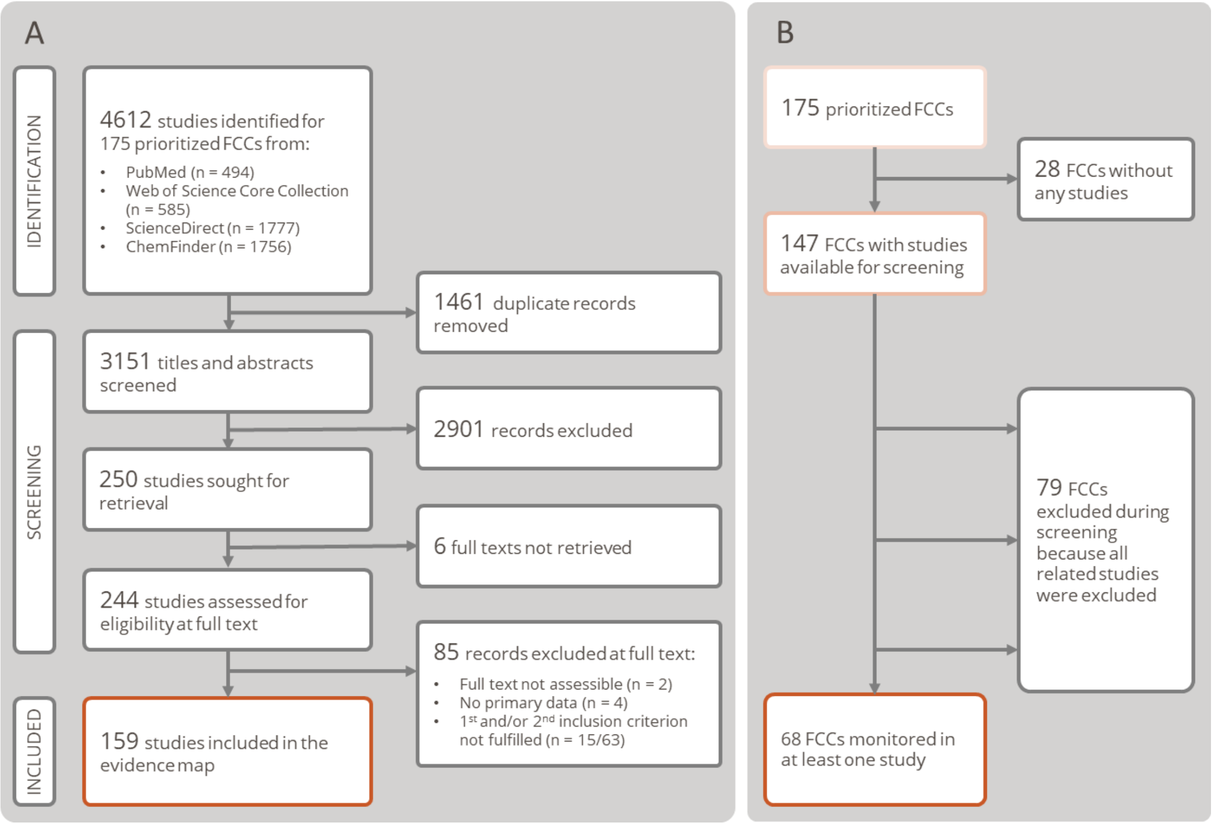

Figure S6. A. Flow diagram for the targeted systematic evidence map on prioritized FCCs that are present in humans, containing the preferred reporting items for systematic reviews and meta-analyses (PRISMA). **B.** Number of FCCs that were included at the different stages of the literature search and screening process.
